# A catalog of microbial genes from the bovine rumen unveils a specialized and diverse biomass-degrading environment

**DOI:** 10.1093/gigascience/giaa057

**Published:** 2020-05-30

**Authors:** Junhua Li, Huanzi Zhong, Yuliaxis Ramayo-Caldas, Nicolas Terrapon, Vincent Lombard, Gabrielle Potocki-Veronese, Jordi Estellé, Milka Popova, Ziyi Yang, Hui Zhang, Fang Li, Shanmei Tang, Fangming Yang, Weineng Chen, Bing Chen, Jiyang Li, Jing Guo, Cécile Martin, Emmanuelle Maguin, Xun Xu, Huanming Yang, Jian Wang, Lise Madsen, Karsten Kristiansen, Bernard Henrissat, Stanislav D Ehrlich, Diego P Morgavi

**Affiliations:** 1 BGI-Shenzhen, Shenzhen 518083, China; 2 China National GeneBank, BGI-Shenzhen, Shenzhen 518120, China; 3 School of Biology and Biological Engineering, South China University of Technology, Guangzhou 510006, China; 4 Laboratory of Genomics and Molecular Biomedicine, Department of Biology, University of Copenhagen, 2100 Copenhagen Ø, Denmark; 5 INRAE, Génétique Animale et Biologie Intégrative, AgroParisTech, Université Paris-Saclay, 78350 Jouy-en-Josas, France; 6 Animal Breeding and Genetics Program, Institute for Research and Technology in Food and Agriculture (IRTA), Torre Marimon, Caldes de Montbui 08140, Spain; 7 CNRS UMR 7257, Aix-Marseille University, 13288 Marseille, France; 8 INRAE, USC 1408 AFMB, 13288 Marseille, France; 9 LISBP, Université de Toulouse, CNRS, INRAE, INSA, 31077 Toulouse, France; 10 Université Clermont Auvergne, INRAE, VetAgro Sup, UMR Herbivores, F-63122 Saint-Genès Champanelle, France; 11 School of Future Technology, University of Chinese Academy of Sciences, Beijing 101408, China; 12 INRAE, Micalis Institute, AgroParisTech, Université Paris-Saclay, 78350 Jouy-en-Josas, France; 13 James D. Watson Institute of Genome Sciences, Hangzhou 310058, China; 14 Institute of Marine Research (IMR), Postboks 1870 Nordnes, 5817 Bergen, Norway; 15 Department of Biological Sciences, King Abdulaziz University, Jeddah, Saudi Arabia; 16 MGP MetaGenoPolis, INRAE, Université Paris-Saclay, 78350 Jouy en Josas, France; 17 Centre for Host Microbiome Interactions, Dental Institute, King's College London, London, UK

**Keywords:** rumen, metagenome, herbivory, carbohydrate-active enzymes, bovine

## Abstract

**Background:**

The rumen microbiota provides essential services to its host and, through its role in ruminant production, contributes to human nutrition and food security. A thorough knowledge of the genetic potential of rumen microbes will provide opportunities for improving the sustainability of ruminant production systems. The availability of gene reference catalogs from gut microbiomes has advanced the understanding of the role of the microbiota in health and disease in humans and other mammals. In this work, we established a catalog of reference prokaryote genes from the bovine rumen.

**Results:**

Using deep metagenome sequencing we identified 13,825,880 non-redundant prokaryote genes from the bovine rumen. Compared to human, pig, and mouse gut metagenome catalogs, the rumen is larger and richer in functions and microbial species associated with the degradation of plant cell wall material and production of methane. Genes encoding enzymes catalyzing the breakdown of plant polysaccharides showed a particularly high richness that is otherwise impossible to infer from available genomes or shallow metagenomics sequencing. The catalog expands the dataset of carbohydrate-degrading enzymes described in the rumen. Using an independent dataset from a group of 77 cattle fed 4 common dietary regimes, we found that only <0.1% of genes were shared by all animals, which contrast with a large overlap for functions, i.e., 63% for KEGG functions. Different diets induced differences in the relative abundance rather than the presence or absence of genes, which explains the great adaptability of cattle to rapidly adjust to dietary changes.

**Conclusions:**

These data bring new insights into functions, carbohydrate-degrading enzymes, and microbes of the rumen to complement the available information on microbial genomes. The catalog is a significant biological resource enabling deeper understanding of phenotypes and biological processes and will be expanded as new data are made available.

## Background

Ruminant production contributes to livelihood and to food and nutritional security in many regions of the world. Milk and meat from ruminants are important sources of protein and micronutrients in the human diet, but often criticized as unsustainable because of the low conversion efficiency of plant feeds into animal foods [1] and their high environmental footprint. However, when the feed conversion efficiency of protein and energy contained in milk and meat is calculated on the basis of the ingestion of human-inedible protein and energy, the output is higher than the input, particularly in forage-based production systems [2, 3]. The transformation of feeds, not suitable for human consumption, into highly nutritious protein and energy products is carried out by gastrointestinal symbiotic microbes, particularly those residing in ruminants’ forestomach, the rumen. Rumen microbes are essential for ruminants, allowing them to thrive on agricultural land not suitable for crops and to consume agricultural byproducts unfit for other livestock species. The enhanced functions provided by the rumen microbiota are key to the characteristic adaptability and robustness of ruminants to cope with nutritional and climatic stresses [4].

Improving our understanding of the rumen microbiota provides opportunities for knowledge-based strategies aiming at enhancing efficacy in ruminant production while minimizing its negative effect on the environment. Great advances in the understanding of rumen microbiota functions have been obtained by extensive genome sequencing of cultured rumen bacteria and archaea (Hungate1000 project) [5] and by assembly of draft genomes from metagenomic data [6–8]. These catalogs of reference genomes and metagenome-assembled genomes (MAGs) provide great insight into the functionality of this ecosystem, but, although extensive, they still do not cover the full bacterial and archaeal diversity present in the rumen [5, 9, 10]. In this study, we used a complementary approach to generate a catalog of unique rumen prokaryotic genes that enabled us to decipher functional potentials of the microbiota as a whole, in particular, the capacity to deconstruct structural carbohydrates from forages, and we explored the effect of feed on the microbiota composition and functions.

## Construction of a bovine rumen prokaryotic gene catalog

To build a bovine rumen prokaryotic gene catalog, we collected samples of total rumen content from 5 Holstein cows and 5 Charolais bulls. To reduce the ecosystem complexity and to improve metagenome assemblies, rumen ciliated protozoa were depleted from the samples before microbial DNA extraction. A total of 1,206 Gb of raw metagenomic sequencing data were generated with a mean of 111 Gb clean data for each animal [11]. This sequencing depth, much greater than that used for gut gene catalogs from humans and other monogastric animals [12–14], was necessary to enable the assembly of the more complex rumen microbiome. After *de novo* assembly, open reading frame (ORF) prediction, and removal of redundancy, 13,825,880 non-redundant genes were obtained with a mean length of 716 bp, and 39% of these genes were complete ORFs (Supplementary Table 1).

Compared to the rumen gene catalog published by Hess et al. [15], the number of non-redundant genes discovered in this study is >5-fold larger; shared genes were in most cases also longer (Supplementary Fig. 1a and b and Supplementary Table 2). Thus, the mapping rate of reads from 77 additional rumen samples obtained in this study and 8 published rumen samples from UK cattle [16] increased from ∼10% using the previous Joint Genome Institute (JGI) catalog [15] to ∼40% (11–51%) (SOAP2, ≥95% identity, Supplementary Fig. 1c and d). This confirms that the representativeness of the rumen catalog was greatly improved, even though the mapping efficiency was still relatively low, as compared to 80% for the human gut microbiome [12]. We also used protein sequences for reduced variability and compared this study to protein sequences (10,703,199) present in the large MAGs dataset of Stewart et al. [7]. The proportion of non-redundant proteins in our catalog was 89%, 53%, and 29% when considered at a similarity cut-off of 100%, 90% or 50%, respectively, whereas for the MAGs dataset proportions were 84%, 39%, and 8% (Supplementary Fig. 1e). Taken as a whole, this information indicates the large diversity present in this ecosystem.

**Figure 1:**
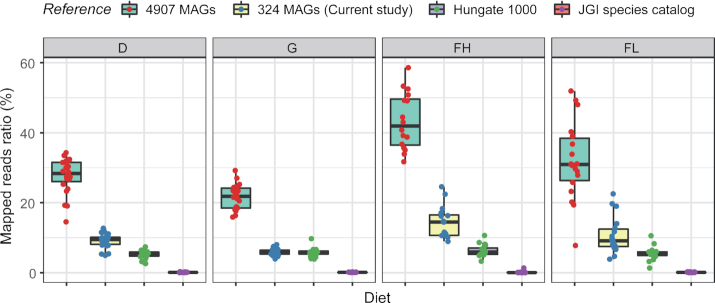
Percentage of total reads in diet groups that mapped to metagenome-assembled genomes (MAGs). Mapping ratios of 77 samples to 4,907 genomes were calculated on the basis of [7]. Mapping ratios of 77 samples to Hungate1000 isolates were calculated on the basis of [5]. Diet groups are corn silage-concentrate (D, n = 23) and grazing (G, n = 20) for Holstein cows and fattening high-starch (FH, n = 16) and fattening low-starch (FL, n = 18) for Charolais bulls.

To compare our data with available genomes, MAGs were constructed on the basis of scaftig abundance profiles and an in-house co-abundance clustering pipeline. We identified 324 MAGs with a mean size of 1.8 Mb (minimum threshold of 1 Mb; see Supplementary Table 3 for more information on these MAGs). More than half (173) were medium-quality (CheckM, ≥50% completion, <10% contamination) and 23 were high-quality drafts (CheckM, >90% completion, <5% contamination) [17]. Except for 1 MAG annotated to the Archaea domain (Euryarchaeota), all were annotated to the Bacteria domain. For the bacterial MAGs, 39% could be annotated to the order level but only 2% (8 MAGs) to the genus level, all belonging to *Prevotella*. To assess their novelty, the rumen MAGs from this work were compared to the Hungate1000 genomes [5] and to the nearly 5,000 MAGs reported from Scottish cattle [6, 7]. Only 24 (7%) of 324 MAGs were similar to genomes from the Hungate1000 project (maximal unique matches index [MUMi] < 0.54), whereas 189 (58%) were similar to Scottish cattle MAGs (Supplementary Table 3). This comparison highlights the novelty represented by draft genomes from metagenomes and the large diversity of the rumen microbiota that is not yet covered in culture collections or MAG collections. In addition, we compared the proportion of mapping ratios of genomes and MAGs to an external dataset obtained from total rumen content samples of 77 cattle from 2 different genetic stocks that were fed diets characteristic of beef and milk production systems. Beef cattle, represented by the Charolais breed, were fed fattening diets high (FH, n = 16) or low (FL, n = 18) in starch and lipids; whereas Holstein dairy cows were fed a corn silage and concentrate diet (D, n = 23) or grazed a natural prairie (G, n = 20) (Supplementary Table 4). The 324 MAGs were present in all 4 diet groups from our validation cohort; ∼10% of reads from the 77 cattle datasets mapped to these MAGs (SOAP2, ≥95% identity). For genomes from the Hungate1000 project [5], which are representative of the diversity of cultured rumen bacteria and archaea, the mapping rate was 5.4%, whereas the mapping rate for the ∼5,000 MAGs collection of Stewart et al. [6, 7] was higher and, depending on the diet, ranged from 21% in G up to 42% in FH. In contrast, only 0.1% of reads mapped to the 15 metagenomic species described by Hess et al. [15] (Fig. 1).

## Analyses

### Comparison of gastrointestinal microbiomes: bovine rumen versus human, pig, and mouse

Genes were taxonomically classified using CARMA3 [18] and compared to genes from the human, mouse, and pig gut catalogs [12–14]. Up to 42.7% of rumen genes could be annotated to known phyla. This value is similar to pig gut (41.3%) but lower than the human (55.9%) and mouse gut metagenomes (59.6%) (Supplementary Fig. 2 and Supplementary Table 5). Firmicutes and Bacteroidetes were predominant in all catalogs, representing 84–94% of assigned genes and in accord with the expected gastrointestinal-associated microbial communities in mammals. For the rumen, however, the proportion of Firmicutes and that of Bacteroidetes was lower and higher, respectively, than for the other 3 catalogs. Other enriched phyla (>2%) in the rumen catalog were the Spirochaetes, Proteobacteria, Euryarchaeota, Actinobacteria, and Fibrobacteres, which, with the exception of Proteobacteria and Actinobacteria in human, were more abundant in the rumen than in the other catalogs (Supplementary Fig. 3). At the genus level, only 8.7% of rumen genes could be annotated, a value similar to that of the other 2 animal catalogs but lower than that of human (16.8%), reflecting a more extensive characterization of human-associated microbes. However, the top 10 enriched genera in the rumen showed distinct abundance patterns compared with the same genera in other catalogs (Supplementary Fig. 4 and Supplementary Tables 5 and 6). These differences in symbiotic microbial genera likely reflect dissimilarities in dietary lifestyles and anatomical localization of the gut fermentation compartment and are indicative of predominant functions, i.e., methane production and plant fiber degradation for ruminants. *Prevotella* was the most abundant rumen genus, with 39% of genus-annotated genes assigned. Other abundant genera were *Treponema, Butyrivibrio, Methanobrevibacter*, and *Ruminococcus*, which were absent or at lower proportions in other catalogs, particularly in the human catalog.

### Carbohydrate active enzymes in the bovine rumen metagenome

The efficient deconstruction of structural plant polysaccharides by symbiotic gastrointestinal microbes is what sets ruminants apart from other livestock species. We have therefore analyzed carbohydrate active enzymes (CAZymes) in the rumen ecosystem to obtain insights into this important function for the nutrition and health of cattle.

Glycoside hydrolases (GHs) and polysaccharide lyases (PLs) are the most relevant classes of CAZymes because they orchestrate the breakdown of plant material and of diverse polysaccharides that are encountered in the rumen ecosystem, i.e., host, fungal, and bacterial glycans. GHs and PLs are classified into sequence-based families (145 GH and 26 PL families [19]) that display a pronounced specificity for a glycan category, thereby offering a functional readout of the degradative power of an ecosystem. The rumen catalog reported here encodes 545,334 CAZymes, of which ∼290,000 have degradative activity, that are affiliated to 114 distinct GH families (97.4%) and 18 PL families (2.6%). These 545,334 CAZymes were compared to GenBank and to the assembled genomes from rumen samples of Stewart et al. [6] (Supplementary Fig. 5). Stewart and co-workers [6] described 69,678 CAZymes with 65–72% identity to other datasets, and 91% of novel CAZymes (defined as having <95% identity to other datasets). Our catalog displays similar features with mean 73% identity to the MAGs of Genbank and Stewart et al. [6] and 89% novel CAZymes (482,759 sequences with <95% identity). It is acknowledged that the larger MAG dataset of Stewart et al. [7] contains >400,000 predicted CAZymes, but these were not yet published when our comparison was made. Also, it is noted that 32,755 degradative CAZymes are present in the Hungate1000 reference genomes [5].

In the rumen catalog, the substrate specificity of the most abundant GH families reflects the prominent glycan sources of herbivores: starch (GH13, GH77, and GH97, by decreasing abundance), pectins, and hemicelluloses (GH43, GH28, GH10, GH51, GH9, and GH78, by decreasing abundance). In contrast, only 1 of the 15 most abundant families, namely, GH25 lysozymes, targets a non-plant substrate (peptidoglycan). Additionally, 3 of the 5 most abundant families (GH3, GH2, and GH5) represent enzymes active on a wide range of substrates, not necessarily from plant origin. Two of these families (GH2 and GH3) contain exo-glycosidases that act on the oligosaccharides produced by depolymerases, a broad function that may explain their abundance.

Dockerins domains (DOCs) are key building blocks of cellulosomes and amylosomes complexes [20, 21]. The DOC sequences are found in modular proteins and help the protein to which they are appended to bind cohesin domains (COHs) found as repeats in large proteins named scaffoldins. This system allows the spatial grouping of numerous binding and enzymatic modules into large assemblies for a synergistic action of their components in the immediate vicinity of the bacterial cell. In the rumen catalog, >12,000 dockerin modules were identified. Intriguingly, some proteins harbored many dockerin modules, up to 13 modules in a single sequence, without any other recognizable functional module. The function of such polydockerin proteins is unknown, and polydockerin proteins were not observed in reconstructed MAGs (maximum of 2 DOCs in a protein). In the literature, dockerin modules, initially detected in cellulosomes, have been investigated in relation to their co-occurrence with CAZymes in these cellulosome complexes [22, 23]. Surprisingly, our analysis of the rumen catalog reveals that only ∼24% of the DOC-containing proteins carry a CAZyme domain. The remaining DOC-containing proteins were subjected to a Pfam domain annotation, which identified proteases (4%) and some lipases (<0.3%), while one-third of DOC-containing proteins are attached to non-catalytic modules, likely involved in the binding of these non-carbohydrate substrates. More importantly, the last third did not have any match to any Pfam domain (Supplementary Fig. 6).

The CAZyme profile in the rumen catalog was compared to the mouse, pig, and human reference gut catalogs [12–14]. Despite important differences in the size of these catalogs, similar trends could be observed in, e.g., the ratio of DOCs or GHs plus PLs to the catalog size, or the most abundant GH families (Supplementary Table 7). The number of distinct GHs/PLs is also very similar, and a detailed analysis highlighted 101 GH families common to all 4 catalogs, while only 5 GH families were specific to a single catalog (Supplementary Fig. 7). These specific families were closely related to the hosts’ diets. In accord with herbivory, 305 GH45 cellulase modules were found in the rumen catalog against none in the human and mouse catalogs, and only 12 for the pig. In contrast, we identified families GH70 and GH68, transglycosidases acting on sucrose, and GH47, processing N-glycan, that are absent in the rumen but present in other catalogs. For instance, 94, 24, and 6 GH70 modules were found in the human, pig, and mouse catalogs, respectively, whereas the rumen had zero occurrence.

**Figure 2:**
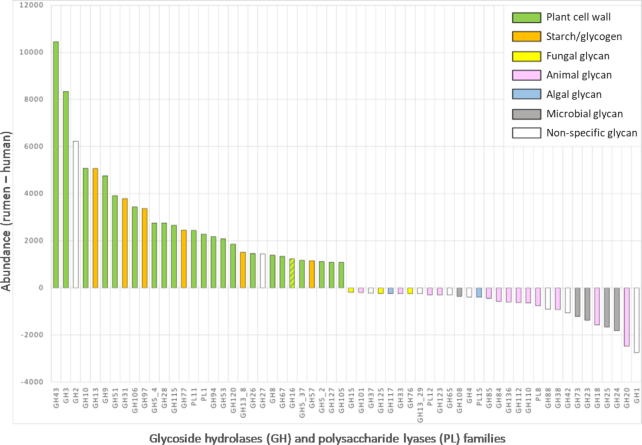
Enrichment or depletion of glycoside hydrolases and polysaccharide lyases in the bovine rumen as compared to human gut. Human counts were normalized to rumen catalog size before comparison.

The specific adaptation of the rumen microbiota to herbivory was confirmed by comparing its GH+PL family counts against the human catalog after normalization (Fig. 2). The most enriched GH families in the rumen are involved in the degradation of plant polysaccharides while the more depleted families of GHs are those degrading animal (host) glycans. These observations are not only in accord with the normal diet of cattle, but they are also in agreement with the absence of a glycoprotein-rich mucus lining of the rumen as opposed to the lower gastrointestinal tract. Finally, we also observed that multiple DOC module duplications seem to be more frequent and intense in the rumen because up to 13 DOC repeats in a single protein were found for the rumen catalog, compared to only 6 in the human, 4 in the pig, and 2 in the mouse catalogs.

CAZyme-encoding genes were also annotated in the 324 MAGs. Remarkably the most abundant families in the MAGs are for plant cell wall breakdown and correspond closely to the most abundant families in the non-redundant catalog. The CAZyme profiles of each generated MAG were thus determined and subjected to a hierarchical clustering analysis (Supplementary Fig. 8) that revealed that the MAGs roughly group together according to their predicted taxonomy, even despite large differences in repertoire size within each phylum. Hereafter, we analyzed in detail several strategies for carbohydrate foraging that have evolved in the different bacterial phyla. Among the predicted Firmicutes, MAGs encoding cellulosomes and amylosomes displayed a readily recognizable profile characterized by the presence of several DOC and COH domains along with several GH families containing cellulases (GH5, GH44, GH48, and GH124) and amylases (GH13 with associated CBM26), respectively. We also identified Bacteroidetes MAGs that contained a few DOC domains, but, interestingly, none of these MAGs contained a recognizable COH domain. The presence of dockerin domains not associated to cohesins in Bacteroidetes MAGs was recently reported in the moose rumen microbiome [8]. The role of the dockerins in Bacteroidetes is unclear, but the conspicuous absence of cohesins suggests that they may not be needed for the assembly of a bona fide cellulosome or that the Bacteroidetes cohesins are so distantly related to their clostridial counterparts that they cannot be recognized.

Confirming previous reports in the literature [24], the largest CAZyme repertoires dedicated to plant degradation were found among the predicted Bacteroidetes members, which represent the majority of the 324 reconstructed genomes. In Bacteroidetes, CAZymes are often grouped in distinct polysaccharide utilization loci (PULs) around *susC* and *susD* marker genes to build up specific depolymerization machineries capable of deconstructing in a synergistic manner even the most complex polysaccharides [25, 26]. In this context, it is interesting to note the clustering of families GH137 to GH143 recently shown to catalyze the breakdown of type II rhamnogalacturonan [25] in the CAZyme profile heat map (Supplementary Fig. 8). Inspection of the predicted PULs in the Bacteroidetes MAGs revealed the presence of degradation machineries dedicated to pectin (type II rhamnogalacturonan), starch, or barley β-glucan (Supplementary Fig. 9).

Other MAGs with distinctive CAZymes were those assigned to Proteobacteria and Fibrobacteres, which despite their small number (8 and 6, respectively) form tight groups. Predicted Proteobacteria were characterized by the presence of families GH84 and GH103 along with an important diversity of GH13 subfamilies. In contrast, the Fibrobacteres show the presence of several families known to degrade cellulose and β-glucans (e.g., GH5, GH45, and GH55). Focusing on CAZymes from *Fibrobacter* spp. present in the catalogue revealed an astonishing strain-level diversity for this genus. We compared the CAZymes present in *Fibrobacter succinogenes* type species [27] against all Fibrobacter CAZymes in the catalog. There were 1,262 hits with ≥90% identity to 135 of the 175 *Fibrobacter succinogenes* CAZymes, whereas only 19 of them had 100% identity with the type strain. Up to 465 and 375 of these genes were differentially abundant in the Holstein and Charolais groups, respectively (Supplementary Table 8). Zooming in on a particularly important endoglucanase enzyme, GH45, reveals its presence in all 77 animals receiving different diets. Animals harbored between 4 and 13 GH45 variants and each gene was present in 25–99% of all animals; however, the type strain, at 79%, was not the variant most commonly present.

### Common functions and influence of diet on the bovine rumen microbiome

**Figure 3:**
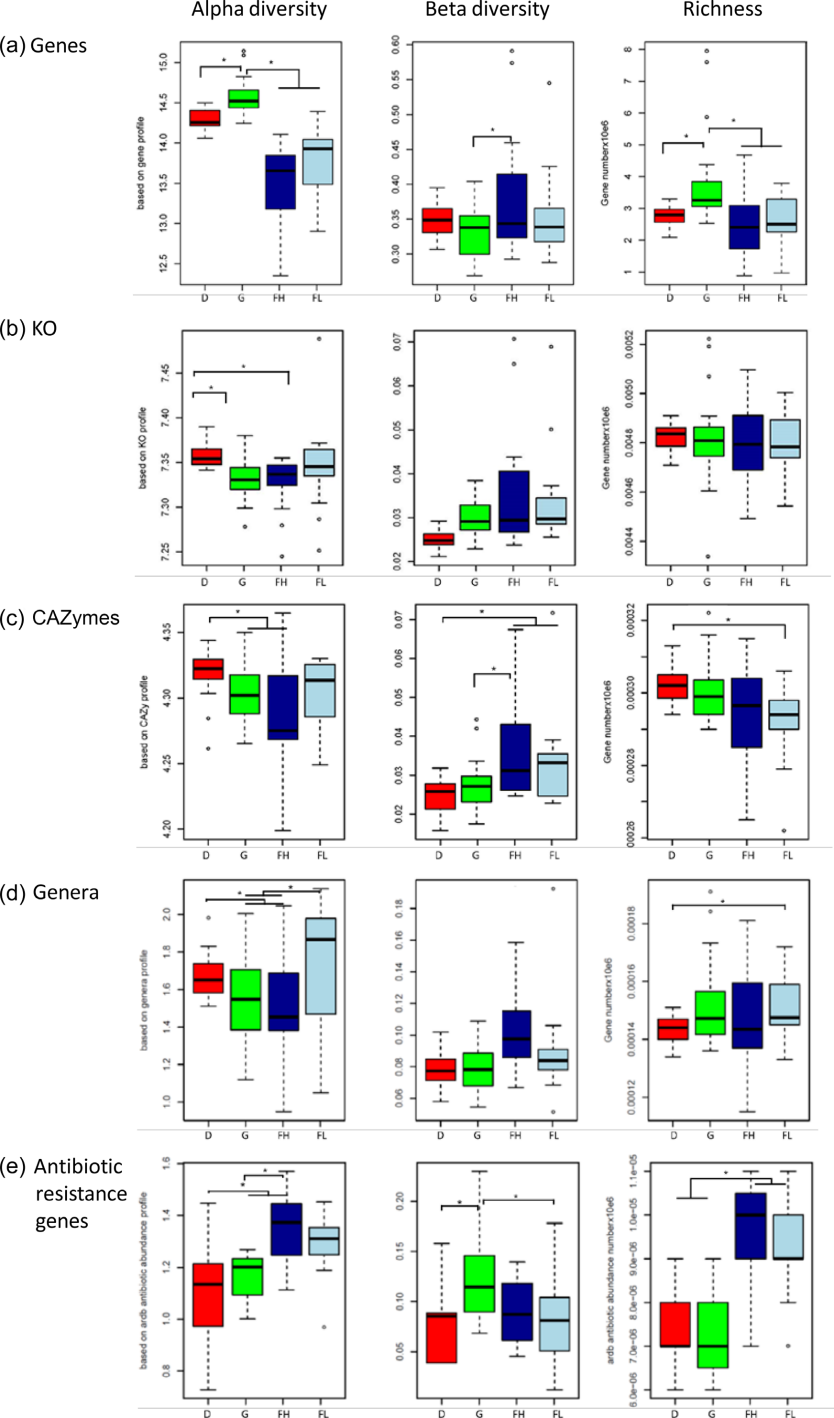
Effect of diet on diversity indexes of the bovine rumen microbiome. Comparison of α-diversity, β-diversity, and richness at gene (a), KEGG orthologs (KO) (b), carbohydrate-active enzymes (CAZymes) (c), genera (d), and antibiotic resistance gene (e) levels among cattle fed dairy (D, red, n = 23), fattening high-starch (FH, dark blue, n = 16), fattening low-starch (FL, light blue, n = 18), and grazing (G, green, n = 20) diets. Asterisk indicates *P* < 0.05. line inside box is median, lower and upper box edges indicate interquantile range (IQR) (25th to 75th percentile, maximum dotted line = Q3 + 1.5*IQR, minimum dottet line = Q1 –1.5*IQR, dots outside line range are outliers.

To investigate how different feeds affected the rumen microbiota in beef and dairy cattle we examined samples from 77 cattle described above. By using this 77-sample dataset, differences in α-diversity were observed between diets at the gene level. Animals fed fresh grass had the highest α-diversity and richness compared to other diets containing conserved feeds. Particularly, animals receiving fattening diets had a lower α-diversity. In contrast, the fattening diet rich in starch and polyunsaturated fatty acids (PUFAs) exhibited the highest β-diversity and/or had the highest disparity in interquartile range (box in the boxplot) for all indices (Fig. 3). The rumen microbiome of animals fed this diet also exhibited the highest dispersion on ordination analyses at the gene level (Supplementary Fig. 10). Such changes, akin to the described Anna Karenina principle [28] for microbiomes, probably reflected divergences in individual microbiome (and host) responses to PUFAs and may underlie a stress response to the diet.

**Figure 4:**
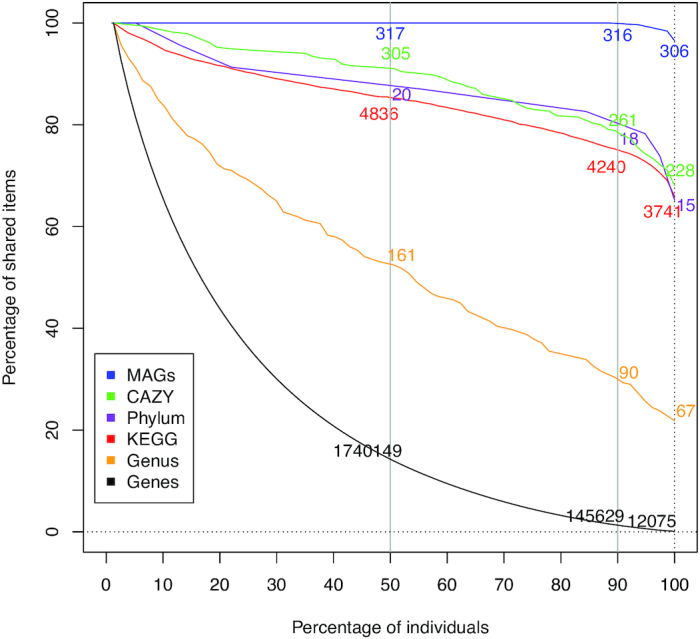
Size of the shared microbiome features among cattle (n = 77) fed 4 different diets for the number of genes, genera, phyla, metagenome-assembled genomes (MAGs), KEGG pathways, and carbohydrate-active enzymes (CAZymes). The percentages of shared items and animals are represented on the *y*- and *x*-axes, respectively. The absolute numbers for each item are indicated at the intercept between the percentages of items and animals at the thresholds of 50%, 90%, and 100%. Only ∼1% of genes were shared by 90% of the cattle, whereas close to 80% of KEGG orthologs and CAZy functions were shared by 90% of the cattle, suggesting gene redundancy for similar functions. Note the presence of most MAGs assembled in this work in 90% of the cattle.

Genes were annotated to known functions (KEGG and Carbohydrate-Active Enzymes Database [CAZy]) and taxonomical information was derived. For functions, there were 43.3% of the genes that could be classified into KEGG orthology and 2.1% assigned to feed carbohydrate degradation. A total of 5,893 unique KEGG orthologs (KOs) and 45,683 unique CAZymes and binding modules were identified. Comparing the annotated genes for KEGG and CAZy functions showed a large overlap among groups, with 91% and 94% of shared genes, respectively (Supplementary Fig. 11). Contrasting with results on overall gene abundance, the highest α-diversity was observed for the corn silage diet group (Fig. 3). To assess the functions encoded by the minimal rumen metagenome, we identified genes and KOs that were shared by all individuals in the group of 77 cattle. We found common sets of non-redundant genes, functions, genera, and MAGs that were shared by all 77 rumen samples (Fig. 4 and Supplementary Fig. 12). The core gene set shared by all animals represented only <0.1% (6,051–12,075 genes depending on the calculation method; see Methods) of the nearly 14 M non-redundant genes in the catalog, whereas ∼63% of the KO functions (∼3,700) were shared, indicating the high redundancy of genes for similar functions. Compared to all annotated KOs, this minimal KO set was substantially enriched in pathways related to metabolism (amino acids, carbohydrate, nucleotides, and metabolism of cofactors and vitamins), cellular processes (motility), and genetic information processing (translation) (Supplementary Fig. 12b). Concerning the diversity of genera found in the different groups, there was also a relatively large overlap. Of 242 genera identified by the taxonomic analysis described above, 182 (75%) were present in all 4 groups but only 67 (28%) were shared by all animals (Fig. 4 and Supplementary Fig. 11). This overlap was maximal for MAGs identified in this study, which were present in virtually all individuals (Fig. 4). The presence of common functions may explain the plasticity of the microbiota and adaptability of ruminants to digest various types of feeds even after sudden dietary changes. To get a better understanding of the functional changes induced by diet in these microbial communities, we analyzed the abundance of genes in the 77-sample dataset for functions, genera, and MAGs. To avoid possible confounding effect of breed and sex, the differential abundance analysis was performed within each breed. For Holstein, greater changes in the relative abundance of genes were observed: ∼43% difference in KEGG and CAZy functions (Supplementary Tables 9–11, and Supplementary Fig. 13a and b). For CAZy, 146 catabolic families indeed exhibited differences in abundance between the corn silage and grazing groups (Supplementary Figs 13a and 14, Supplementary Table 10). Most of the differences related to functions were due to increases in the relative abundance of genes in cows fed the corn silage diet rather than the presence of different genes. Notwithstanding, the greatest contrast was observed for families targeting fructans and sucrose that were more abundant in the grazing group, particularly for family GH32 (*P* = 7.6E−12) whose higher abundance could be related to the high contents of sucrose and fructans in grasses [29, 30] included in the grazing diet. The other CAZy families differing in abundance were all more abundant in the corn silage diet group. Interestingly, these results highlight the ability of ruminal bacteria to be equally capable of using glycans from plants as well as from microbial origin such as bacterial peptidoglycans, bacterial exopolysaccharides, and fungal cell walls. Corn silage, the main constituent of the diet, is a fermented feed with an abundant epiphytic microbiota composed of exopolysaccharide-producing lactic acid bacteria, fungi, and yeasts [31, 32]. Accordingly, the CAZome of the corn silage diet group was oriented towards degradation of starch, a nutrient abundant in corn silage and practically absent in the grazing diet. Forty-two CAZy families targeting plant cell wall polysaccharides were also overabundant in the corn silage diet group. This could reflect the diversity of fiber structures that ruminal bacteria have to face when cows are fed with such a diversified diet in terms of plant fractions and botanical origins (whole corn plant and soybean meal in the corn silage diet against a natural prairie, composed predominantly of grasses, in the grazing diet). Finally, the overabundance of CAZy families targeting animal glycans in the silage-fed cohort was striking because no glycoprotein-rich mucus is secreted in the bovine rumen as opposed to the lower gastrointestinal tract [33]. It is possible that this difference reflects that CAZy families targeting animal glycans harbor numerous enzymes that are not fully characterized and may be able to act on plant or even fungal glycans, which contain a panel of osidic constituents that are very similar to that of animal glycans. Enzyme promiscuity may indeed confer metabolic flexibility and an ecological advantage to certain microbes in the gut ecosystem.

For genera and MAGs, up to 44% (106 genera) and 58% (188 MAGs) of the total detected were differently abundant in the microbial communities of the 2 cows’ groups (Supplementary Table 12 and Supplementary Fig. 13c and d). *Fibrobacter* and *Ruminoccocus* were more abundant in the corn silage diet group whereas *Prevotella, Butyrivibrio*, and *Methanobrevibacter* were more abundant in the grazing group.

For Charolais on fattening diets differing in starch content, <5% differences were observed in the abundance of genes for functions or genera. Only 8 CAZy families exhibited differences in abundance between the 2 Charolais groups, the differences in abundances being less significant than for the Holstein groups (*P* = 0.004) (Supplementary Figs 13a and 14, Supplementary Table 10). The absence of marked variations in the abundance of glycoside-degrading enzymes between the 2 fattening diets indeed reflects their similar composition. The differences in starch content were not great enough to drastically affect the carbohydrate-harvesting functions of the ruminal microbiota, at least at the gene level. Similarly, smaller differences in the abundance of genera and MAGs were detected between these 2 diets (Supplementary Table 12, Supplementary Fig. 13c and d).

Metadata collected on the Holstein and Charolais animals were analyzed using the adonis function from Vegan [34] (Supplementary Table 13). Diet had a significant effect on metagenome gene distribution, particularly in the Holstein group (*r*^2^ = 0.68, *P* = 0.0001), but also variables such as live weight, feed intake, and rumen volatile fatty acids were significant. Protozoal numbers were also a significant variable explaining the distribution of genes in the metagenome of animals, underpinning their importance as key members of the rumen ecosystem and modulators of the prokaryotic community.

### Antibiotic resistance genes

The spread of antibiotic resistance pathogens in the environment is a great concern in public health. Livestock species are a known reservoir of antibiotic resistance genes (ARGs) [35]. Information from ruminants is predominantly from the fecal microbiome [36], and although the importance of the rumen microbiome has also been highlighted [37, 38], data on the rumen resistome is still fragmented. As an example of the useful information that can be retrieved from a gene catalog, we evaluated the presence of ARGs in the rumen microbiome as previously reported [13]. Forty-two ARGs encoding resistance to 27 antibiotics were detected in the catalog. The most abundant resistances were to tetracycline and bacitracin, with Charolais animals harboring globally a higher proportion of these genes (Supplementary Fig. 15), probably reflecting the effect of diet [37]. It is noted that antibiotics as growth promoters were never used on these animals. In both the bovine rumen and the porcine gut [13], the most abundant ARGs confer resistance to tetracycline and bacitracin. The diversity of ARGs is low compared to pig feces, where resistance to up to 52 antibiotics was reported, even in farms with no use of growth-promoting antibiotics [13]. Similar to this study, tetracycline resistance was reported as highly abundant in the rumen; otherwise prevalence of resistance to other antibiotics varies between studies [38, 39]. Although the methodologies used to detect ARGs could play a role in these differences [37–39], it is probable that variation in the rumen resistome may differ between countries and regions because it can reflect decades of exposure since antibiotics started to be used in farms.

## Discussion

Ruminants are extraordinary bioreactors, engineered by nature to use recalcitrant plant biomass—a renewable resource—as feedstock for growth and for production of useful products. This ability is a microbial attribute that was important in domestication and that today has a renewed interest due to human population increases, resource scarcity, and climate change issues. The reference gene catalog from the rumen microbiota reported here is a useful resource for future metagenomics studies to decipher the functions and interactions of this complex ecosystem with feeds and the host animal. Comparison with human, mouse, and pig gut catalogs shows the distinct character and potential of the rumen ecosystem. As opposed to the microbiome of single-stomached animals including humans, the rumen microbiome harbors a plethora of genes coding for glycoside hydrolases (CAZymes) that degrade structural polysaccharides. Information on these enzymes that deconstruct biomass plant material and are essential for transforming recalcitrant feeds into meat and milk is also useful for the design of improved processes for the biofuel industry [40, 41].

The type of diet modulated as expected the abundance of genes and the metagenome profile of individuals. However, >90% of genes coding for functions (KO and CAZy) were shared among animals receiving different diets. This large functional diversity might be the key that allows ruminants to feed on a variety of dietary sources and to adapt to seasonal or production-imposed dietary changes. The 13.8 M gene catalog produced in this work, despite being significantly larger than gut bacterial catalogs from other species [12–14], does only partially cover the diversity present in the rumen microbiome, indicating the higher complexity of this ecosystem. The catalog needs to be expanded with additional data, particularly the inclusion of ciliated protozoa and fungi, to reflect the overall diversity. Nevertheless, this catalog and the 324 uncultured assembled genomes are an important instrument to characterize and elucidate the biological functions of the rumen microbiome. This information is essential to enhance the sustainability of ruminant production.

## Methods

This study was conducted using the animal facilities at the French National Institute for Agricultural Research (INRAE) in Theix and Bourges, France. Procedures on animals used in this study complied with the guidelines for animal research of the French Ministry of Agriculture and all other applicable national and European guidelines and regulations.

### Rumen sampling

Total rumen content samples from 10 animals (5 Charolais bulls and 5 Holstein cows) used for deep sequencing metagenome were taken at the experimental slaughterhouse of the INRAE Centre Auvergne-Rhône-Alpes (Supplementary Table 4). Total rumen content samples from 77 animals were also collected. These 77 animals, from 2 different genetic stocks, were fed diets characteristic of beef and milk production systems. Beef cattle, represented by the Charolais breed, were fed fattening diets high (n = 16) or low (n = 18) in starch and lipids, whereas Holstein dairy cows were fed a corn silage and concentrate diet (n = 23) or grazed a natural prairie (n = 20) (Supplementary Table 4). Rumen samples from these animals were also collected at the experimental slaughterhouse except for the grazing group. Cows from this latter group were fitted with rumen cannula, and samples were taken from live animals.

### Sample handling and DNA extraction

The 10 rumen samples used for deep sequencing were depleted of eukaryotes using washing and centrifugation steps. Rumen contents were filtered through a 400-µm nylon monofilament mesh. The filtrate was centrifuged at 300*g*, 5 min, to decant protozoa, and the supernatant (fraction A) was stored at 4°C. Fifty grams from the filtered rumen content retentate were mixed with 100 mL of anaerobic phosphate-buffered saline (PBS), mixed manually for 5 min by gentle inversion, centrifuged at 300*g*, 5 min, to decant protozoa, and the supernatant, passed through a 100-µm filter (fraction B), was stored at 4°C. The pellet was mixed with 75 mL anaerobic, ice-chilled 0.15% Tween 80 in PBS and incubated on ice for 2.5 h to detach microbes attached to feed particles. At the end of the incubation, contents were vortexed for 15 s and centrifuged at 500*g*, 15 min. The supernatant (fraction C) was mixed with fraction B and 50 mL of fraction A and centrifuged at 20,000*g*, 20 min, 4°C. The supernatant was decanted and the microbial pellet was exposed to an osmotic shock to lyse any remaining eukaryote (mainly protozoal) cells followed by an endonuclease treatment. Briefly, the pellet was suspended in water (Millipore Waters Milli Q purification unit) and incubated for 1 h at room temperature followed by DNase treatment (Benzonase, Novagen) as described [42]. The suspension was filtered through a 10-µm monofilament textile, collected by centrifugation as before, suspended in an appropriate volume of PBS, and stored at −80°C until DNA extraction. DNA was extracted following the method described by Yu and Morrison [43]. Samples from 77 animals were extracted directly from whole rumen contents using the same extraction method.

### DNA library construction and sequencing

Paired-end metagenomic libraries were constructed and sequenced following Illumina HiSeq2000 instructions. Quality control and bovine DNA removal (by aligning reads to *Bos taurus* genome Btau_4.0 [44]) for each sample were independently processed using the MOCAT pipeline as previously described [10]. On average, 111.3 Gb of high-quality reads were generated for each of the 10 deep sequencing samples and 3.43 Gb (median ∼2.5 Gb) for each of the 77 samples (Supplementary Table 4). The averaged proportion of high-quality reads among all raw reads from each sample was 92.29%.

### Public data use

The 5 public rumen microbial datasets used in this study include (i) a cow rumen microbiome sequenced at the U.S. Department of Energy's JGI in 2011 , which consists of 268 Gb of metagenomics sequences, 2,547,270 predicted genes, and 15 uncultured microbial genomes assembled from the cow rumen [15] (NCBI accession No. SRA023560); (ii) 8 rumen metagenomics samples from beef steers [16] (European Bioinformatics Institute [EBI], PRJEB10338); (iii) 501 rumen microbial genomes from the Hungate1000 Project (Integrated Microbial Genomes [IMG], JGI Proposal ID: 612/300816); (iv) 913 draft MAGs from Scottish cows’ rumen (EBI, PRJEB21624); and (v) 4,907 draft MAGs from Scottish cattle rumen (Aberdeen Angus, Limousin, Charolais, and Luing) that were available at the time of the analysis (EBI, PRJEB31266). Three public gut microbial gene datasets from human [12, 45], mouse [14, 46], and pig [13] (EBI, PRJEB11755) were also collected.

### Construction of the rumen microbial gene catalog

High-quality reads from 10 deep sequenced samples were processed in MOCAT toolkit [10] including *de novo* individual assembly (SOAPdenovo v1.06 [47], -K 47). The assembled contigs with length ≥500 bp were used for gene prediction (MetaGeneMark [48], –M 100 –A) and redundant genes were removed (CD-HIT [49], ≥95% identity and ≥90% overlap, -n 8 -d 0 -g 1 -T 6 -G 0 -aS 0.9 -c 0.95), resulting in a non-redundant rumen microbial gene catalog containing 13,825,880 genes (Supplementary Table 1).

### Evaluation of current rumen microbial gene catalog

To assess the representative of our rumen gene catalog, we used the rumen gene catalog published to date by Hess et al. [15]. First, the genes with gaps were filtered as follows: genes were broken where they meet the “*N*” base and a subset for each interrupted gene was obtained, retaining only the longest sub-gene as representative of the original gene. A total of 2.46 M genes without gaps were obtained, termed “JGI-2011-gene-catalog,” and used for the following analysis (Supplementary Table 2).

Furthermore, 13.83 M genes from the present study and 2.46 M genes from JGI were pooled together to identify shared genes using CD-HIT with ≥95% identity and ≥90% overlap [49]. The comparison of shared gene length between the 2 catalogs (represented genes and redundant genes) was conducted according to Li et al. [12]. Length discrepancies between shared genes in both catalogs that were <10% were considered as similar and those >10% were considered as longer or shorter. In a similar way, 10,703,199 unique genes were identified from the MAG database of Stewart et al. [7] and compared to the 13.83 M genes from this study. The comparison was made through BLASTP queries at 100%, 90%, and 50% identity levels using Diamond [50] (settings –sensitive -k 1 –max-hsps 1 -o matches.m8). High-quality reads of 77 rumen samples in the present study and 8 UK cattle rumen samples [16] were aligned against the present gene catalog (13.83 M genes) and JGI-2011-gene-catalog (2.46 M genes) using SOAP2 (≥95% identity) [51]. The read mapping ratio was calculated as the number of mapped reads to the total reads in each sample.

### Gene catalog annotation

Taxonomic assignments of genes from rumen, mouse, pig, and human guts were performed using CARMA3 [18] on the basis of BLASTP [52] (V2.2.24) against the NCBI-NR database (v20130906 for rumen, mouse, pig guts; v20160219 for human gut) (Supplementary Table 5). Microbiota from these 4 species were compared at different taxonomic levels. Functional annotation based on the KEGG database was performed using an in-house pipeline [12]. Annotation of the CAZymes of each catalog was performed by comparing the predicted protein sequences to those in the CAZy database and to hidden Markov models built from each CAZy family [53], following a procedure previously described for other metagenomics analyses [8]. Comparisons to the NCBI-NR and Stewart et al. [6] datasets were made through BLASTP queries. Because predicting CAZymes with automatic tools such as used by Stewart et al. [6] might not be as exhaustive as CAZy curation, we compared our annotated CAZymes to the full protein datasets to allow an exact estimation of the repertoire novelty. For each annotated CAZyme, we recorded the best BLAST hit, based on bit-score, and computed the average identity percentages compared to each and combined datasets. Similar to the procedure used by Stewart et al. [6], we considered as “novel” a protein without any hit above a threshold of 95% of identity. To allow a direct comparison of the results, annotation of ARGs was done as previously reported in the pig metagenome catalogue [13] by using the ARDB database [54].

### Construction relative abundance profiles of genes, KOs, ARGs, and CAZymes

The gene profiles of 77 rumen samples were generated by aligning high-quality clean reads to the current 13.83 M gene catalogue (SOAP2, ≥95% identity) [51]. Gene relative abundance was estimated as described previously [55]. The relative abundance of each KEGG orthologous group (KO), ARGs, and CAZymes was calculated from the abundance of its genes [12].

### Characterization of total and minimal metagenome

We computed the total and shared number of genes, KOs, and CAZy functions present in random combinations of *n* individuals (with n = 2–77, 100 replicates per bin) [55]. Furthermore, we used a permutation test to identify the second-level KEGG functions that were significantly enriched or depleted in the minimal KO set compared with the total KO set. We first calculated the contribution of second-level functions using the following formula:

$$\begin{equation*}
{p_{ij}} = \frac{{{f_{ij}}}}{{\mathop \sum \nolimits_j {f_{ij}}}},\,\,{P_j} = \frac{{\mathop \sum \nolimits_i {p_{ij}}}}{N},
\end{equation*}$$

where ${f_{ij}}$ is the number of second-level function *j* from the KO *i*, ${p_{ij}}$ is the relative contribution of second-level function *j* in the KO *i, N* is the number of KOs in the KO set, and ${P_j}$ is the relative contribution of function *j* in the KO set.

Randomly sampling 999 times in all annotated KO sets simulated the distribution of each function. Calculating the position of this function contribution ratio of the minimal KO set under the distribution of all annotated KO sets, a *P*-value of <0.01 was regarded as significant (Supplementary Fig. 12b).

### Construction of MAGs and taxonomic assignment

To recover the draft bacterial and archaeal genomes from the 10 deep sequenced samples, we developed an in-house pipeline that comprises 3 steps as indicated in Supplementary Fig. 16 and described below.

1. *Construction of scaftig-linkage groups*

We first performed scaffolding of contigs using paired-end Illumina reads (SOAPdenovo v1.06) and constructed scaftigs by extracting the contiguous sequences that lack unknown bases (Ns) in each scaffold [55]. We then generated a scaftig abundance profile by aligning high-quality clean reads from 77 rumen samples to assembled scaftigs from samples [51]. Scaftig relative abundance was determined using the same method applied for gene abundance [51]. The highly co-abundance–correlated scaftigs from each deep sequencing sample were binned into scaftig-linkage groups (SLGs) using the previously described pipeline [51] with modified parameters as follows: an edge was assigned between 2 scaftigs sharing Pearson correlation coefficient >0.7, and the minimum edge density between a join was set as 0.99. A total of 745 preliminary SLGs with length >1 Mb were generated for further analysis.

2. *Filtering of preliminary SLGs based on GC content and assembly outputs*

For all preliminary SLGs, we then examined their specificity by plotting the GC content versus reads aligned depth of each scaftig. In this step, 520 SLGs containing a sole GC cluster were treated as “qualified” and retained for Step 3. For the remaining 225 SLGs, 184 presented a scattered GC distribution and were discarded whereas the 41 SLGs containing 2 or more GC clusters were further processed. First, those SLGs with scaftig N50 <2,000 bp were considered as too fragmented and discarded. Then, multiple GC clusters in remaining SLGs were separated by DBSCAN [56] (Eps ≤ 0.10, MinPts ≥ 49). After splitting and filtering, we retained 55 “qualified” SLGs that had a coverage depth >20×.

3. *Reconstruction of metagenome-assembled genomes*

To improve the completeness and remove the redundancy of multiple metagenome assemblies from 10 deep sequencing samples, we performed hierarchical clustering for these 575 qualified SLGs based on their scaftigs nucleotide identity calculated by MUMi [57]. The MUMi distance between 2 SLGs (a and b) was defined as follows:

$$\begin{equation*}
{\rm{MUMi\ }} = \left({1 - \displaystyle\frac{{{L_{\mathrm{unmap}{\rm{\ }}\mathrm{length}{\rm{\ }}\mathrm{of}{\rm{\ }}a}} + {L_{\mathrm{unmap}{\rm{\ }}\mathrm{length}{\rm{\ }}\mathrm{of}{\rm{\ }}b}}}}{{{L_{\mathrm{total}{\rm{\ }}\mathrm{length}{\rm{\ }}\mathrm{of}{\rm{\ }}a}} + {L_{\mathrm{total}{\rm{\ }}\mathrm{length}{\rm{\ }}\mathrm{of}{\rm{\ }}b}}}}}\right)/M,
\end{equation*}$$

where *M* = 2 × min (*L*_total length of *a*_, *L*_total length of *b*_)/(*L*_total length of *a*_, *L*_total length of *b*_), *L*_total length of *a*_ is the length of SLG a, and *L*_unmap length of *a*_ is the length of unmapped sequence compared with SLG b. The threshold for generating a species-level MAG was set at 0.54 for MUMi distance value, as previously suggested [58]. There were 218 qualified SLGs that could not be clustered with other SLGs and were defined as singleton MAGs. The remaining 357 qualified SLGs were clustered into 105 candidate MAGs. We performed overlap-based assembly on the scaftigs for each of these 105 candidate MAGs, respectively, using Phrap with default parameters. To get reliable contiguous sequences for each candidate MAG, the overlaps between 2 scaftigs <500 bp were considered as unreliable and re-broken.

The 105 reconstructed candidate MAGs were further examined using GC patterns using the same method mentioned in Step 2 above. Eighty of the 105 candidate MAGs containing a sole GC cluster were retained as combined MAGs. The remaining 25 candidate MAGs containing 2 or more clusters were split into sub-MAGs using the same method mentioned in Step 2 above. To preserve the most comprehensive genomic information for these sub-MAGs, sequences from each sub-MAG were aligned back to their original SLGs. If the sub-MAG covered ≥90% sequences of its original SLG, it would be retained as a revised MAG. Otherwise, its original SLG will replace the corresponding sub-MAGs and be considered as a revised MAG. This splitting step finally obtained 31 revised MAGs.

After filtering the total sequence size of 218 singleton MAGs, 80 combined MAGs, and 31 revised MAGs with the criterion of >1 Mb, we finally obtained 324 MAGs for rumen microbiota including 224 singleton MAGs and 100 combined MAGs (Supplementary Table 14). We used the same pipeline described above for the gene catalog for the ORF prediction and taxonomic annotation of MAG genes. We used CheckM [59] to estimate the completeness, contamination, and heterogeneity of metagenomic species (Supplementary Table 14). MAGs were assigned a taxonomic level annotation if >50% of their genes were assigned at a given taxonomic level (including genes with no match) (Supplementary Table 15). The MAG relative abundance of 77 rumen samples was calculated from the relative abundance of its aligned genes.

### Quality assessment and taxonomic annotation of MAGs

CheckM software [59] was used to calculate the completeness and contamination of these MAGs. The median percentage of completeness was high, at 62.5% with a low, 2.6% contamination. The combined MAGs showed higher completeness but also slightly higher levels of contamination and strain heterogeneity than singleton MAGs (Supplementary Figs 17 and 18). Taxonomic annotation for rumen MAGs was performed using CARMA3 on the basis of BLASTP against the NCBI-NR database (v20130906) and compared with MAGs from pig and mice (Supplementary Table 15, Supplementary Fig. 19).

### Comparisons between 324 MAGs and public rumen microbial genomes

High-quality reads of 77 rumen samples were aligned against the assemblies of the 324 MAGs in the present study, of the nearly 5,000 MAGs from Scottish cattle [6, 7], of the 409 genomes of microbes isolated from rumen (Hungate 1000; Supplementary Table 16) [5], and of the 15 MAGs from JGI using SOAP2 (≥95% identity) [51]. Mapping ratios of 77 rumen samples to the rumen microbial genome collections from the above studies were calculated as number of mapped reads to number of total reads. Whole-genome similarities between the presrent 324 MAGs and published rumen microbial genomes were calculated using MUMmer. MAGs showing MUMi values <0.54, a suggested threshold for generating a species-level MAG [58], with published rumen microbial genomes were considered as novel MAGs (Supplementary Table 3).

### Cluster distribution by diet at species level

The relative MAG abundance profile (matrix of 324 × 77) obtained above was analyzed to highlight differences induced by diet. As we found when coverage of a MAG is <0.1 the depth of this MAG is close to 0 (Supplementary Fig. 20). This result was caused by the noise and is non-conducive to the MAG clustering. Therefore, when the coverage value was less than this threshold value, we set the value of depth equal to 0.

### Ordination and differential abundance analyses

Breed and diet distribution were visualized in ordination analyses based on 2D non-metric multidimensional scaling [60]. Dissimilarity between pairs of samples was calculated using the Bray-Curtis dissimilarity index [61]. The Vegan R package [34] was also used to estimate the diversity indexes corresponding to richness, α- (Shannon index), and β-diversity (Whittaker). Permutational multivariate analysis of variance (PERMANOVA) test using the adonis function from Vegan [34] was employed to identify host-covariates from the 77 samples (metadata table in Li et al. [11]) that may contribute to the overall pattern of the rumen microbiome structure. Significance levels were determined after 10,000 permutations, and the multiple comparison tests were performed using false discovery rate (FDR).

The relative abundance of the 13,825,880 non-redundant genes was collapsed into taxonomic (phylum and genus) and functional levels (KEGG and CAZy). Procrustes rotation analysis was performed to compare the ordinations obtained at different levels. Identified KOs were mapped to KEGG and visualized using the Interactive Pathway Explorer (iPath2.0) web-based tool [62]. To estimate a core, the overlapping number of genera, CAZymes, and KOs between Holstein and Charolais breeds was compared.

To avoid confounding factors such as sex, breed, and age, the differential abundance analysis was performed within breeds. Therefore, for each breed, diet comparison was performed on the basis of a zero-inflated Gaussian mixture model as implemented in the fitZig function of the metagenomeSeq R package [63]. Correction for multiple testing was done, and the cut-off of the differential abundance was set at FDR ≤ 0.05.

## Availability of Supporting Data and Materials

Metagenomic sequencing data generated in this study have been deposited in the EBI database under the accession code PRJEB23561. The data of assembled scaftigs, the rumen gene catalog, the rumen MAG catalog, and the abundance profile tables generated in this study are available in the *GigaScience* database, GigaDB [11].

## Additional Files

**Table S1**. Summary information of the bovine rumen prokaryotic gene catalog described in this study.

**Table S2**. Summary information of the bovine rumen gene catalog described by Hess et al. (2011) [15] pre- and post-filtering.

**Table S3**. Whole-genome similarity between MAGs from this work, Hungate1000 genomes [5], and MAGs from Stewart et al. [6, 7].

**Table S4**. Metagenomic data production from bovine rumen samples.

**Table S5**. Number of genes and taxonomic annotation information for bovine rumen and human, pig, and mouse feces gene catalogs.

**Table S6**. Detailed annotation information at genus level for rumen, pig, and mouse catalogs.

**Table S7**. Proportion of carbohydrate active enzymes (CAZymes) in the rumen microbiome of cattle and gut microbiomes of human, pig, and mouse.

**Table S8**. Effect of diet on the abundance of CAZy genes assigned to *Fibrobacter succinogenes* in the bovine rumen metagenome. Gene counts of the differentially abundant genes in the D and G samples (spreadsheet “Holstein”), and in the FL and fattening high starch + linseed diet (FH) samples (spreadsheet “Charolais”). Similarity of 1,262 CAZy Fibrobacteres genes enumerated in the catalogue to *Fibrobacter succinogenes* S85.

**Table S9**. Number of differentially abundant genera, MAGs, CAZymes, and KOs within each breed detected by differential abundance analysis.

**Table S10**. Effect of diet on the abundance of CAZy families in the bovine rumen metagenome. Gene counts of the differentially abundant families of glycoside hydrolases, polysaccharide lyases, family 35 of glycosyl transferases, carbohydrate-binding modules, dockerins, and cohesins in the dairy cow (D) and grazing (G) diet samples (sheet “Holstein”), and in the fattening low-starch diet (FL) and fattening high starch + linseed diet (FH) samples (sheet “Charolais”). The targeted substrates are indicated for each family.

**Table S11**. Effect of diet on the abundance of KO in the bovine rumen metagenome.

**Table S12**. Effect of diet on the abundance of genera and MAGs in the bovine rumen metagenome.

**Table S13**. Vector fitting table.

**Table S14**. Evaluation of assembly quality for 324 rumen MAGs.

**Table S15**. Taxonomic annotation for MAGs catalog of rumen, pig, and mouse by CARMA3.

**Table S16**. List of microbial genomes from the Hungate1000 project used in this work.

**Figure S1**. (a) Identity of present study genes compared to Hess et al. [15]. (b) Differences in gene length between studies. Purple indicates that the length of genes is longer in the present study; blue, the length of genes is similar; pink, the length of genes is shorter in the pres study. (c) Percentage of total reads in the present study (n = 77 samples) in 4 different diet groups (D = dairy cow, G = grazing diet, FH = fattening high-starch + linseed diet, and FL = fattening low-starch diet) that could be mapped to the present study gene catalog and JGI 2011 gene catalog [1]. (d) Percentage of total reads in unrelated studies of 8 UK cattle samples [2] that could be mapped to the present study gene catalog and JGI 2011 gene catalog. (e) Diagram of unique and shared (intersection of circles) proteins in the present study compared to proteins present in MAGs from Stewart et al. [6].

**Figure S2**. The composition of 4 gene catalogs at different taxonomic annotation levels.

**Figure S3**. The composition of 4 gene catalogs at phylum level.

**Figure S4**. The composition of 4 gene catalogs at genus level.

**Figure S5**. CAZyme sequence diversity of our dataset against reference datasets. Distribution of identity percent obtained after best BLAST hits taking as queries the CAZymes in our catalog and searching for similarities in Genbank or Stewart et al. (n = 913) [7] datasets, alone or combined.

**Figure S6**. Functional domains present in dockerin-containing proteins from the rumen gene catalog.

**Figure S7**. Venn diagram of glycoside hydrolase families present in the gene catalogs from rumen, human, pig, and mouse gut microbiota.

**Figure S8**. Hierarchical clustering of 324 metagenome-assembled genomes (MAGs) according to their CAZyme profiles. Rows display the 324 MAGs with their identifiers on the right and predicted taxonomical phyla on the left (color-coded according to the top right box). Columns display the abundance of glycoside hydrolase and polysaccharide lyase families (colored according to the top left scale). Clustering was computed using the average-linkage method and Spearman rank correlation. Four groups with specific CAZyme signatures are highlighted by red rectangles and labeled: (A) for Proteobacteria having specific GH13 subfamilies 19/32/37, and families GH84, GH103, and GH119; (B) for Fibrobacteres with many cellulases from families GH5, GH45, and GH55 and associated CBM11 and CBM30; (C) Firmicutes (likely from *Ruminoccocus* genus) with cellulosomal apparatus (dockerins and cohesins associated to various cellulases in famillies GH5, GH44, GH48, or GH124); and (D) Bacteroidetes with recently discovered families GH137–GH141 required to fully process the highly complex pectin component rhamnogalacturonan II.

**Figure S9**. Examples of polysaccharide utilization loci (PULs) found in bovine rumen metagenomic species (MAG) and their similarity to experimentally validated PULs with known target substrates. Rearrangements in PUL organization are indicated by grey shapes or segments linking gene homologs. Conservation between homologous proteins is illustrated by identity percentages obtained by BLASTP alignments.

**Figure S10**. Non-metric multidimensional scaling (NMDS) gene counts by diet type (n = 77). The 77 samples were from 4 different diet groups: D = dairy cow (n = 23), G = grazing diet (n = 20), FH = fattening high-starch + linseed diet (n = 16), and FL = fattening low-starch diet (n = 18).

**Figure S11**. Overlapping number of genera, CAZymes, and KEGG Orthology (KO) across diets (n = 77). The 77 samples were from 4 different diet groups: D = dairy cow (n = 23), G = grazing diet (n = 20), FH = fattening high-starch + linseed diet (n = 16), and FL = fattening low-starch diet (n = 18).

**Figure S12**. Size of the shared microbiome features among cattle fed 4 different diets using different calculation methods than that of Fig. 2 for comparison (see Methods section). (a) The size of the minimal metagenome at KO levels (cut-off = 1 × 10^−7^). There are 5,893 functionally annotated KO levels, and a minimal set of 3,706 functions was found for the 77 individuals sampled. (b) Change in the rate of all annotated KO and minimal KO in the second function level. (c) The size of the minimal metagenome at CAZyme levels (cut-off = 1 × 10^−7^). There are 1,974 functionally annotated CAZyme levels, and the minimal set found was of 439 functions. (d) The size of the minimal metagenome at gene levels (cut-off = 1 × 10*^-^*^7^); 6,051 genes was the minimal set for the 77 individuals sampled.

**Figure S13**. (**A**) Cluster distribution in Holstein and Charolais breeds based on CAZy families. Changes in differentially abundant families for each breed are shown in the tables below the graph (arrows indicate higher abundance). The complete list of families is available in Supplementary Table 10. (**B**) Cluster distribution in Holstein and Charolais breeds based on KEGG orthology. Changes in the 10 most differentially abundant KO for each breed are shown below the graph. The complete list is available in Supplementary Table 11. (**C**) Cluster distribution in Holstein and Charolais breeds based on microbial genera. Changes in differentially abundant genera are shown in the tables below the graph (arrows indicate higher abundance). The complete list is available in Supplementary Table 12. (**D**) Cluster distribution in Holstein and Charolais breeds based on MAGs. The complete list is available in Supplementary Table 12. In all panels, for Holstein, red label indicates dairy cow (D) diet and green indicates grazing (G) diet. For Charolais, dark blue indicates fattening high-starch + linseed (FH) diet and light blue indicates fattening low-starch (FL) diet.

**Figure S14**. Effect of diet on the abundance of CAZy families in the bovine rumen metagenome. Difference of abundance of CAZy families, clustered by substrate categories, between dairy cow diet (D, n = 23) and grazing diet (G, n = 20) samples (A), and between fattening low-starch diet (FL, n = 16) and fattening high-starch + linseed (FH, n = 18) samples (B). Only the most significant differentially abundant catabolic CAZy families (glycoside hydrolases, polysaccharide lyases, and family 35 of glycosyl transferases, of which the members are able to cleave osidic linkages of starch and glycogen by phosphorolysis) and their associated non-catalytic modules were taken into account (adjusted *P*-value < 0.05). Color legend: pale purple, fungal glycans; dark purple, bacterial glycans; pale green, pectin; medium green, cellulose; dark green, hemicellulose. Polyspecific families containing members acting on various types of substrates are assigned to “other substrates.”

**Figure S15**. Prevalence of antibiotic resistance genes (ARGs) in the 77-sample cohort. (a) Relative abundances (log_10_ scale) of ARGs (top) and antibiotic types (bottom) found in each individual. In every column on the *x*-axis each dot represents an animal, with colors according to diets: grazing (G; green), dairy (D; red), fattening high-starch + linseed (FH; dark blue), and fattening low-starch (FL; light blue). The higher the vertical position of the dots on the *y*-axis, the higher the relative abundance of the ARGs. (b) Non-metric multidimensional scaling (NMDS) biplot of the ARGs showing a separation between beef (FH and FL diets) and dairy breeds (D and G diets). Grey diamonds represent the individual ARG ordination onto the 2D space, with the names of a subset of ARGs given in black.

**Figure S16**. Diagram describing the approach used for the construction of rumen metagenome-assembled genomes

**Figure S17**. Percentage of completeness and contamination in 324 MAGs. The median completeness and contamination reached 62.48% and 2.62%, respectively (see Supplementary Table 14), showing that the 324 MAGs have high completeness and low contamination.

**Figure S18**. Completeness, contamination, and strain heterogeneity in singleton (blue) and combined (green) MAGs. (a) Proportion of completeness between combined and singleton MAGs. Most MAGs show a completeness >40%. (b) Frequency of contamination between combined and singleton MAGs. Contamination in most MAGs is 0, the rest of the MAGs' contamination is mainly due to combined MAGs. (c) Proportion of strain heterogeneity between combined and singleton MAGs. Strain heterogeneity in most singleton MAGs is 0, whereas most combined MAGs have a strain heterogeneity >70%. (d) When MAG completeness is >40%, then most MAGs' contamination is <10%. (e) When the MAGs' contamination >10%, the contamination (mostly combined MAGs) basically comes from strain heterogeneity.

**Figure S19**. The distribution of MAGs from the bovine rumen and pig and mice feces metagenomes at different taxonomic levels (a). MAG distribution at phylum (b) and genus (c) level.

**Figure S20**. Relationship between coverage and depth for MAGs. Each point represents 1 MAG in 1 of the 77 samples. Histogram in the top and right-hand side indicates the fraction of coverage and depth, respectively. When the coverage is <10%, then the value of depth is close to 0.

## Abbreviations

ARDB: Antibiotic Resistance Genes Database; ARG: antibiotic resistance gene; BLAST: Basic Local Alignment Search Tool; bp: base pairs; CAZy: Carbohydrate-Active Enzymes Database; CAZyme: carbohydrate active enzyme; COH: cohesin domain; D: dairy diet based on corn silage and concentrate; DOC: dockerins domain; EBI: European Bioinformatics Institute; FH: fattening diet high in starch and lipids; FL: fattening diet low in starch and lipids; FDR: false discovery rate; G: natural prairie diet (grazing); Gb: gigabase pairs; GC: guanine-cytosine; GH: glycoside hydrolase; IMG: Integrated Microbial Genomes; INRAE: French National Institute for Agricultural Research; JGI: Joint Genome Institute; KEGG: Kyoto Encyclopedia of Genes and Genomes; KO: KEGG ortholog; MAG: metagenome-assembled genome; Mb: megabase pairs; MUMi: maximal unique matches index; NCBI: National Center for Biotechnology Information; ORF: open reading frame; PBS: phosphate-buffered saline; PL: polysaccharide lyase; PUFA: polyunsaturated fatty acid; PUL: polysaccharide utilization locus; SLG: scaftig-linkage group.

## Ethics Approval

Procedures with cattle were conducted in accordance with the guidelines for animal research of the French Ministry of Agriculture and applicable European guidelines and regulations for experimentation with animals (Certificate of Authorization to Experiment on Living Animals No. 0 04495 and ethics committee notification 10 726–2 016 062 616 304 407 V4).

## Competing Interests

The authors declare that they have no competing interests.

## Funding

This work had the financial support of the Animal Physiology and Livestock Systems Division of INRAE, the INRAE metaprogramme Meta-omics of Microbial Ecosystems (MEM), and BGI-Shenzhen. This study was supported by the National Science and Technology Major Project of the Ministry of Science and Technology of China (grant No. 2017ZX10303406). The work on CAZymes was supported by a European Union's Seventh Framework Program (FP/2007/2013)/ European Research Council (ERC) Grant Agreement 322820 to B.H. Work by Metagenopoliswas supported from grant ANR-11-DPBS-0001. Y.R.C.'s salary was funded by the European Union, in the framework of the Marie-Curie FP7 COFUND People Programme, through the award of an AgreenSkills' fellowship (grant No. 267196) linked to the MEM METALIT project.

## Authors' Contributions

Junhua Li, H.Z., S.D.E., and D.P.M. designed the work and managed the project. Junhua Li and H.Z. designed the analyses and analyzed and interpreted the sequencing data. Junhua Li, H.Z., S.D.E., G.P.V., B.H., N.T., V.L., Y.R.C., J.E., and D.P.M. wrote the manuscript. G.P.V., B.H., N.T., and V.L. conducted data analysis on CAZymes and were involved in the interpretation of data. Y.R.C. and J.E. conducted integrative data analysis, implemented ARG analysis, and were involved in the interpretation of data. M.P. and C.M. were involved in the implementation of animal studies, samples, and metadata collection. Z.Y., H.Z., F.L., S.T., and F.Y. performed data analyses, constructed and annotated the MAG catalog, and compared the MAGs with other published datasets. W.C., B.C., and Jiyang Li performed data analyses and constructed and annotated the gene catalog. J.G. contributed to the experimental development and discussion for filtering rumen samples. M.P., E.M., X.X., H.Y., L.M., and J.W. contributed to text revision and discussion. K.K. interpreted the data and revised the manuscript. D.P.M. coordinated the project. All authors approved the submitted versions and agree to be accountable for all aspects of the work.

## Supplementary Material

giaa057_GIGA-D-19-00154_Original_Submission

giaa057_GIGA-D-19-00154_Revision_1

giaa057_GIGA-D-19-00154_Revision_2

giaa057_GIGA-D-19-00154_Revision_3

giaa057_Response_to_Reviewer_Comments_Original_Submission

giaa057_Response_to_Reviewer_Comments_Revision_1

giaa057_Response_to_Reviewer_Comments_Revision_2

giaa057_Reviewer_1_Report_Original_Submission Amanda Warr -- 6/19/2019 Reviewed

giaa057_Reviewer_1_Report_Revision_1 Amanda Warr -- 12/10/2019 Reviewed

giaa057_Reviewer_2_Report_Original_Submission Marina Martinez Alvaro, PhD -- 12/10/2019 Reviewed

giaa057_Supplemental_Files
