# Supplementary material for: A catalog of microbial genes from the bovine rumen unveils a specialized and diverse biomass-degrading environment
Source: Gigascience. 2020 May 30;9(6):giaa057. doi: 10.1093/gigascience/giaa057 (PMC7260996; doi:10.1093/gigascience/giaa057)
Supplement: giaa057_Supplemental_Files [file giaa057_supplemental_files.zip › Suppl_Figures & tables_GigaSci_rev3_April2020_clean.docx]

SUPPLEMENTARY FIGURES and TABLES

[Results 3](#_Toc22209482)

[Constitution of a bovine rumen prokaryotic gene catalog 3](#_Toc22209483)

[Supplementary Figure 1. Comparison with other rumen gene catalogs 3](#_Toc22209484)

[Comparison of gastrointestinal microbiomes: bovine rumen versus Human, pig and mouse 4](#_Toc22209485)

[Supplementary Figure 2. The composition of four gene catalogs at different taxonomic annotation levels 4](#_Toc22209486)

[Supplementary Figure 3. The composition of four gene catalogs at Phylum level 5](#_Toc22209487)

[Supplementary Figure 4 The composition of four gene catalogs at genus level 6](#_Toc22209488)

[Carbohydrate active enzymes in the bovine rumen metagenome 7](#_Toc22209489)

[Supplementary Figure 5 Mapped ratio on different ‘species’ catalog 7](#_Toc22209490)

[Supplementary Figure 6. Fonctional domains present in dockerin-containing proteins from the rumen gene catalog. 8](#_Toc22209491)

[Supplementary Figure 7. Venn diagram of glycoside hydrolase families 9](#_Toc22209492)

[Supplementary Figure 8. Hierarchical clustering of 324 metagenomics species (MAG) according to their CAZyme profiles 10](#_Toc22209493)

[Supplementary Figure 9. Examples of Polysaccharide Utilization Loci (PULs) 11](#_Toc22209494)

[Common functions and influence of diet on the bovine rumen microbiome 12](#_Toc22209495)

[Supplementary Figure 10. Non-metric multidimensional scaling (NMDS) gene counts by diet type (n= 77) 12](#_Toc22209496)

[Supplementary Figure 11. Overlapping number of Genus, CAZy and KEGG Orthology (KO) across diets (n= 77) 13](#_Toc22209497)

[Supplementary Figure 12. Size of the shared microbiome features among cattle fed four different diets 14](#_Toc22209498)

[Supplementary Figure 13. Cluster distribution by diet 16](#_Toc22209499)

[Supplementary Figure 14. Effect of diet on the abundance of CAZy families in the bovine rumen metagenome. 20](#_Toc22209500)

[Antibiotic resistance genes 21](#_Toc22209501)

[Supplementary Figure 15. Antibiotic resistance genes 21](#_Toc22209502)

[Methods 22](#_Toc22209503)

[Construction of metagenomic species (MAG) and taxonomic assignment 22](#_Toc22209504)

[Supplementary Figure 16. Diagram describing the approach used for the construction of rumen metagenomics clusters 22](#_Toc22209505)

[Supplementary Figure 17. Percentage of 324 MAGs completeness and contamination. 23](#_Toc22209506)

[Supplementary Figure 18. Completeness, Contamination and Strain heterogeneity in MAGs. 24](#_Toc22209507)

[Quality assessment and taxonomic annotation of MAGs 25](#_Toc22209508)

[Supplementary Figure 19. The distribution of MAG from the bovine rumen and pig and mice feces metagenomes at different taxonomic levels. 25](#_Toc22209509)

[Supplementary Figure 20. Relationship between coverage and depth for MAG 26](#_Toc22209510)

[Supplementary Table 1. Summary information of the bovine rumen prokaryotic gene catalog described in this study 27](#_Toc22209511)

[Supplementary Table 2. Summary information of the bovine rumen gene catalog described by Hess et al. (2011) [1] pre- and post-filtering 27](#_Toc22209512)

[Supplementary Table 3. Excel file “Suppl_Table_3_comparison_genomes” 28](#_Toc22209513)

[Supplementary Table 4. Excel file “Suppl_Table_4_10+77samples” 28](#_Toc22209514)

[Supplementary Table 5. Excel file “Suppl_Table_5_catalogs_rumen_pig_mouse” 28](#_Toc22209515)

[Supplementary Table 6. Detailed annotation information at genus level for rumen, pig and mice catalogs 28](#_Toc22209516)

[Supplementary Table 7. Excel file “Supplementary Table 7_CAZy-counts_human_rumen_mouse_pig_catalogs” 29](#_Toc22209517)

[Supplementary Table 8. Excel file “Supplementary Table 8_DA_Fibrobacter_CAZy” 29](#_Toc22209518)

[Supplementary Table 9. Differential abundance analysis 30](#_Toc22209519)

[Supplementary Table 10. Excel file “Suppl_Table_10_DA_CAZy” 30](#_Toc22209520)

[Supplementary Table 11. Excel file “Suppl_Table_11_DA_KO” 30](#_Toc22209521)

[Supplementary Table 12. Excel file “Supplementary Table 12_DA_Genus_MAG” 30](#_Toc22209522)

[Supplementary Table 13. Vector fitting Table 31](#_Toc22209523)

[Supplementary Table 14. Excel file “Suppl_Table_14_animal_information” 32](#_Toc22209524)

[Supplementary Table 15. Excel file “Supplementary Table 15_Assessment of 324_MAG” 32](#_Toc22209525)

[Supplementary Table 16. Excel file “Supplementary Table 16_rumen-pig-mice_MAG_annotation” 32](#_Toc22209526)

[Supplementary Table 17. Excel file “Suppl_Table_17_Hungate_genomes” 32](#_Toc22209527)

## Results

Constitution of a bovine rumen prokaryotic gene catalog

Supplementary Figure 1. Comparison with other rumen gene catalogs

c)


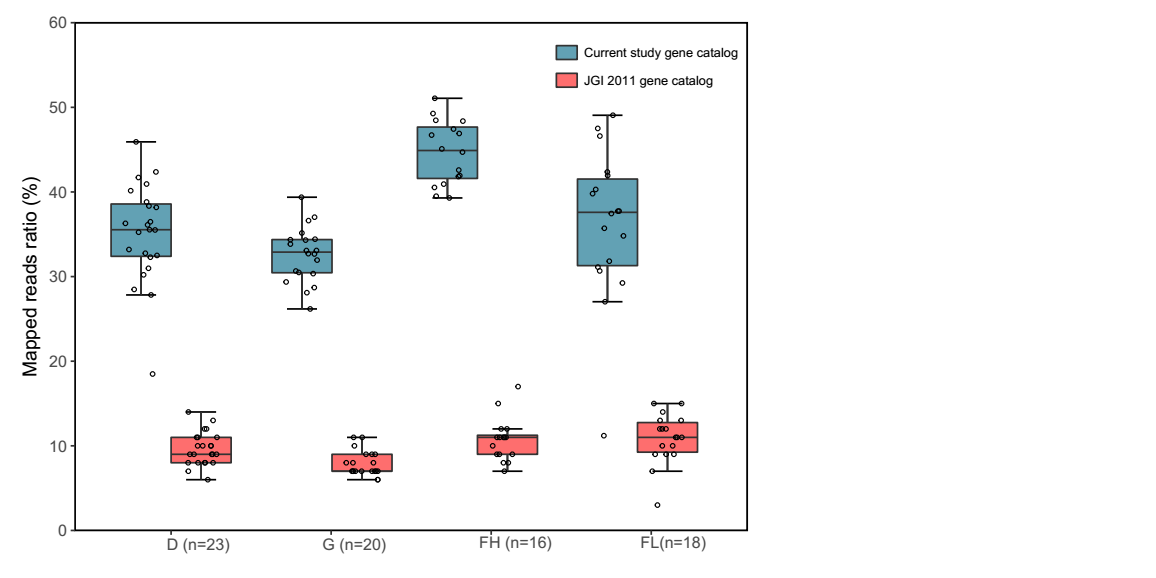


d)


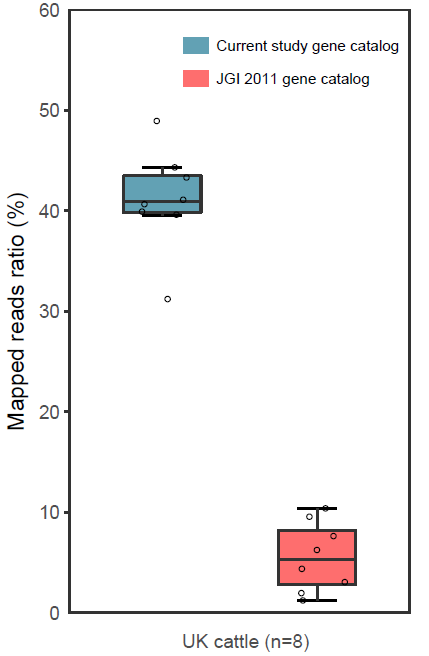


e)


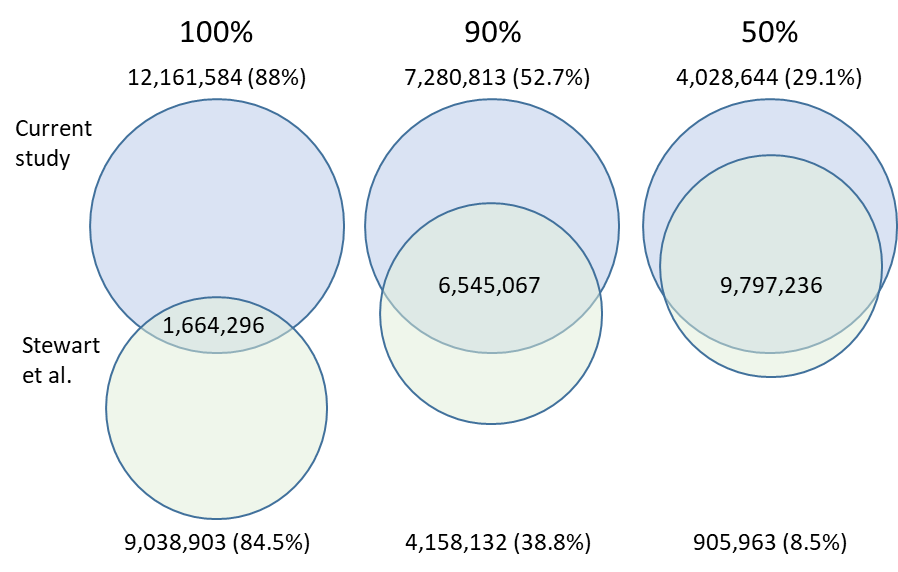


Supplementary Figure 1. (a) Identity of current study genes compared to Hess et al. [[1](#_ENREF_1)]. (b) Differences in gene length between studies. Purple, the length of genes is longer in the current study; Blue, the length of genes is similar; Pink, the length of genes is shorter in the current study. (c) Percentage of total reads in the current study (n=77 samples) in four different diet groups (D= dairy cow, G= grazing diet, FH= fattening high starch + linseed diet, and FL= fattening low starch diet). that could be mapped to current study gene catalog and JGI 2011 gene catalog [[1](#_ENREF_1)]. (d) Percentage of total reads in unrelated studies of 8 UK cattle samples [[2](#_ENREF_2)] that could be mapped to the current study gene catalog and JGI 2011 gene catalog. (e) Diagram of unique and shared (intersection of circles) proteins in the current study compared to proteins present in MAGs from Stewart et al. [[3](#_ENREF_3)]

Comparison of gastrointestinal microbiomes: bovine rumen versus Human, pig and mouse

Supplementary Figure 2. The composition of four gene catalogs at different taxonomic annotation levels

Supplementary Figure 2. The composition of four gene catalogs at different taxonomic annotation levels

Supplementary Figure 3. The composition of four gene catalogs at Phylum level

Supplementary Figure 3. The composition of four gene catalogs at Phylum level

Supplementary Figure 4 The composition of four gene catalogs at genus level

Supplementary Figure 4. The composition of four gene catalogs at genus level

Carbohydrate active enzymes in the bovine rumen metagenome

Supplementary Figure 5 Mapped ratio on different ‘species’ catalog


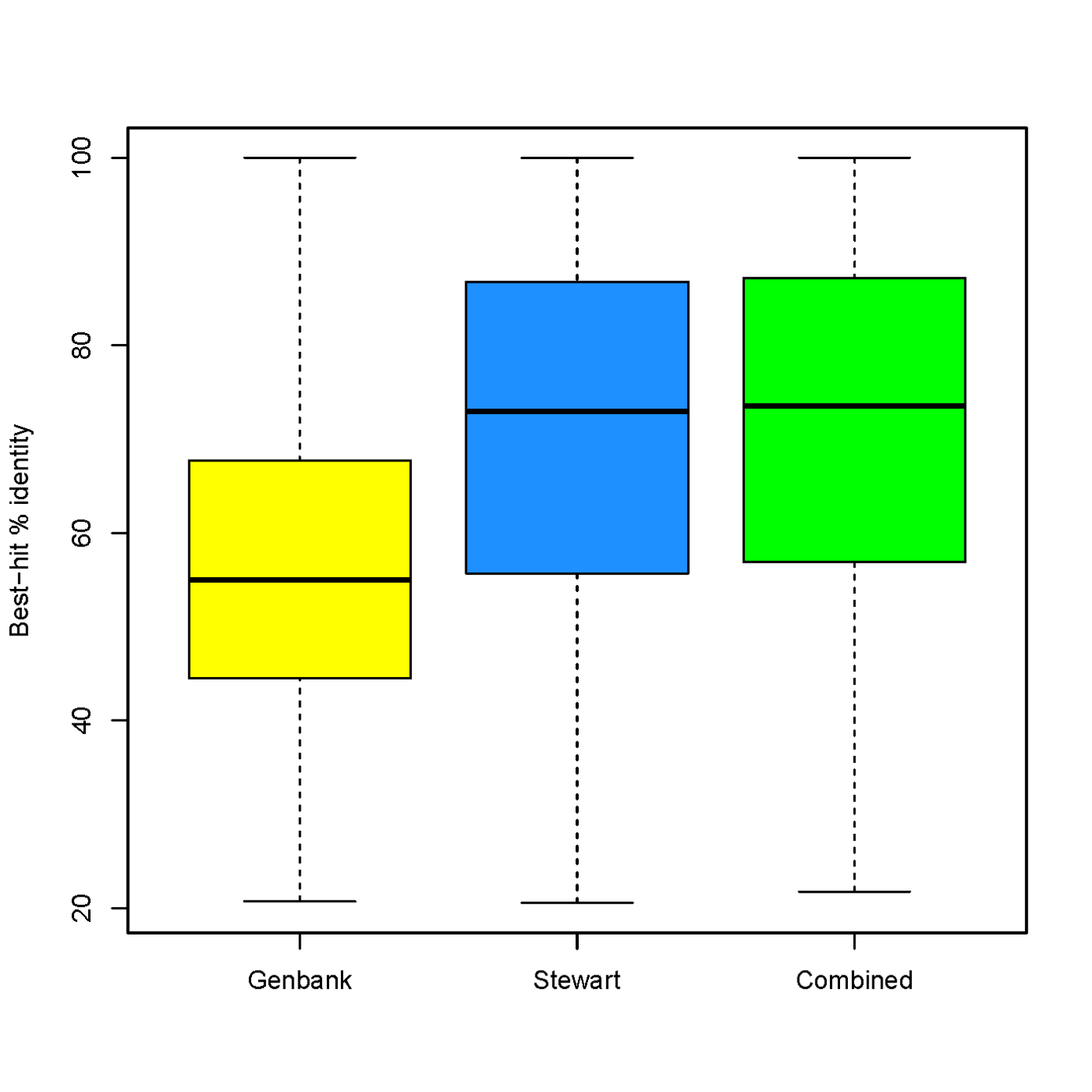


Supplementary Figure 5. CAZyme sequence diversity of our dataset against reference datasets.
Distribution of identity percent obtained after best blast hits taking as queries the CAZymes in our catalog and searching for similarities in Genbank or Stewart et al. (n=913) [[4](#_ENREF_4)] datasets, alone or combined.

Supplementary Figure 6. Functional domains present in dockerin-containing proteins from the rumen gene catalog.


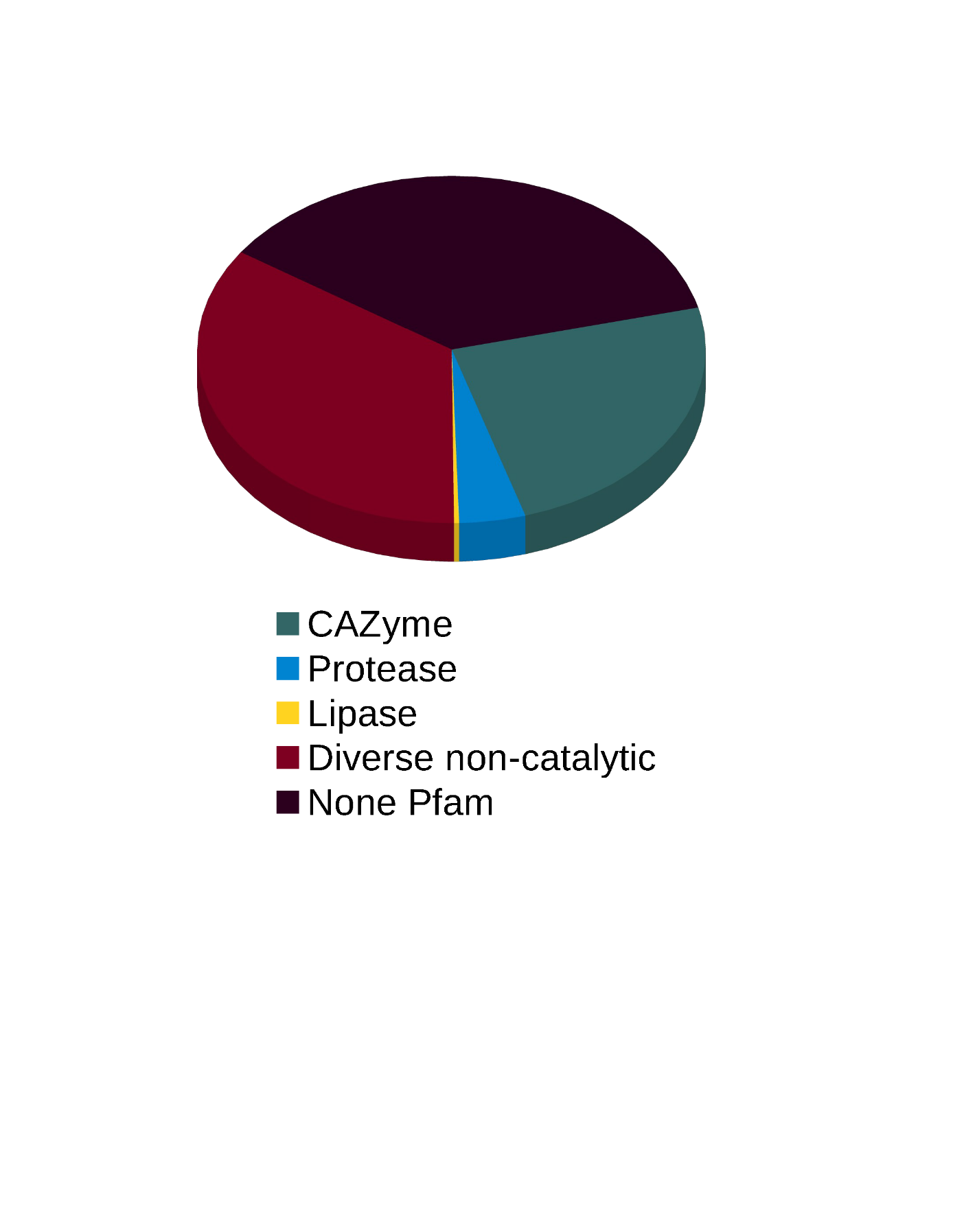
 Supplementary Figure 6. Functional domains present in dockerin-containing proteins from the rumen gene catalog.

Supplementary Figure 7. Venn diagram of glycoside hydrolase families


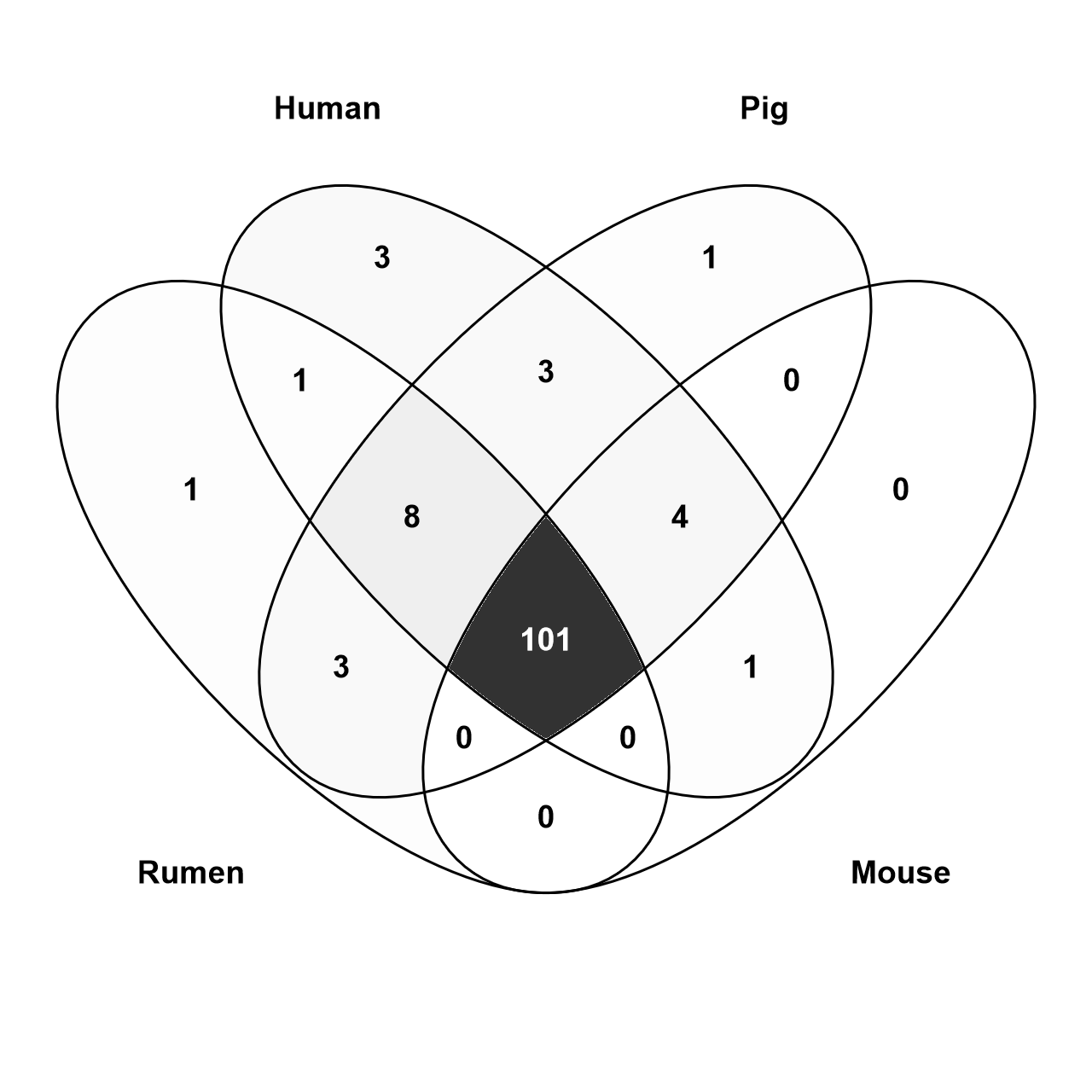


Supplementary Figure 7. Venn diagram of glycoside hydrolase families present in the gene catalogs from rumen, human, pig and mouse gut microbiota

Supplementary Figure 8. Hierarchical clustering of 324 metagenome-assembled genomes (MAG) according to their CAZyme profiles
See “suppFig8-HighRes.pdf” for high resolution image


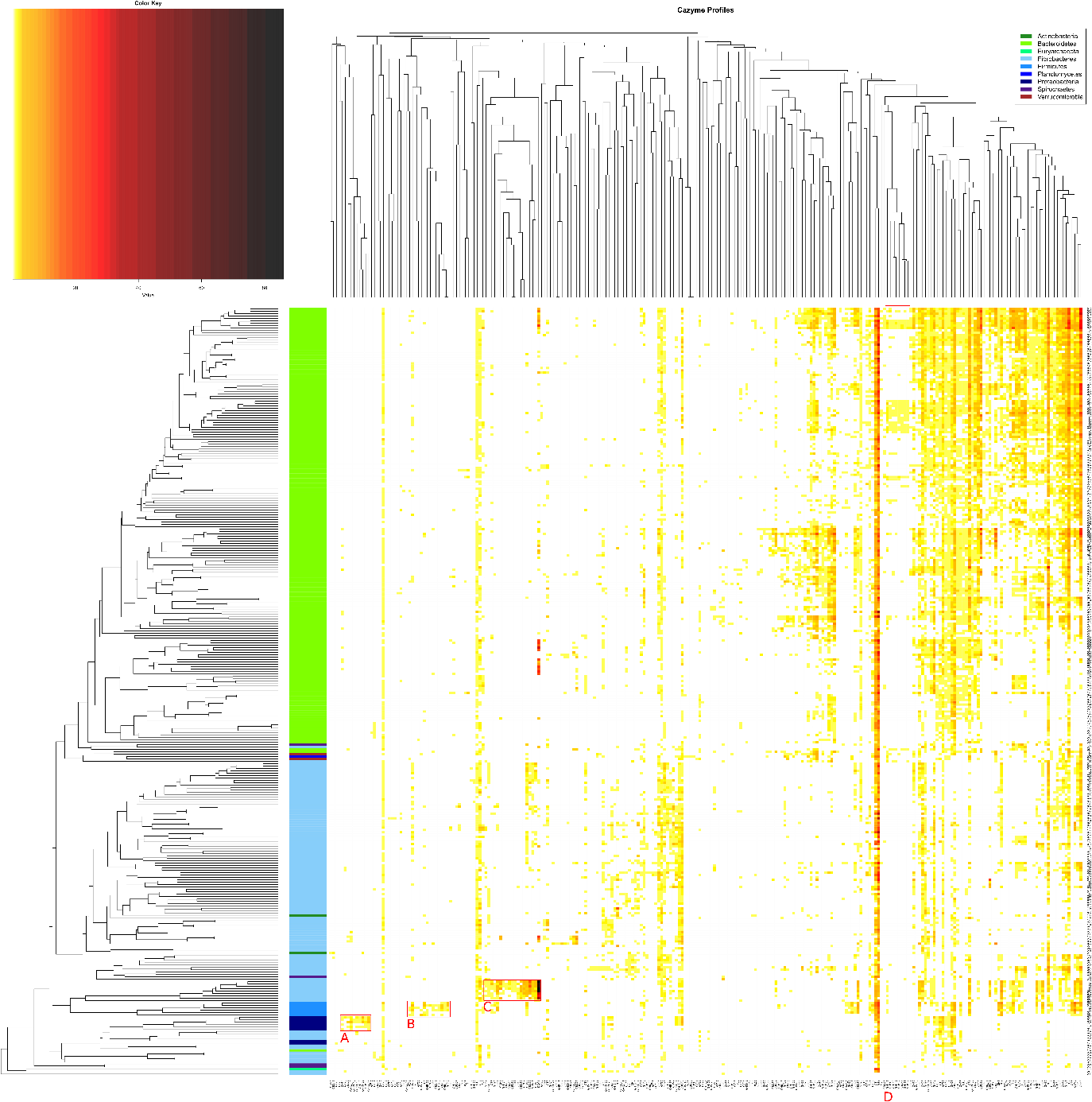


Supplementary Figure 8. Hierarchical clustering of 324 metagenome-assembled genomes (MAG) according to their CAZyme profiles.

Rows display the 324 MAGs with their identifiers on the right and predicted taxonomical phyla on the left (color-coded according to the top-right box). Columns display the abundance of glycoside hydrolase and polysaccharide lyase families (colored according to the top-left scale). Clustering was computed using the average-linkage method and Spearman’s rank correlation. Four groups with specific CAZyme signatures are highlighted by red rectangles and labelled: (A) for *Proteobacteria* having specific GH13 subfamilies 19/32/37, and families GH84, GH103 and GH119; (B) for *Fibrobacteres* with many cellulases from families GH5, GH45 and GH55 and associated CBM11 and CBM30; (C) *Fimicutes* (likely from Ruminoccocus genus) with cellulosomal apparatus (dockerins and cohesins associated to various cellulases in famillies GH5, GH44, GH48 or GH124); and (D) *Bacteroidetes* with recently discovered families GH137 to GH141 required to fully process the highly complex pectin component rhamnogalacturonan II

Supplementary Figure 9. Examples of Polysaccharide Utilization Loci (PULs)


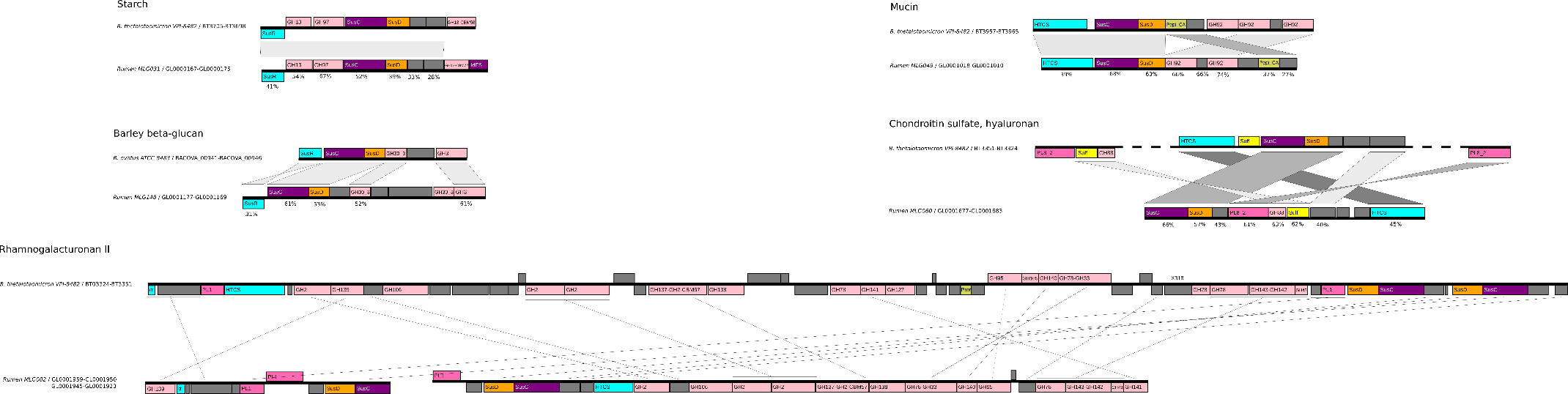


Supplementary Figure 9. Examples of Polysaccharide Utilization Loci (PULs) found in bovine rumen metagenomic species (MAG) and their similarity to experimentally validated PULs with known target substrates. Rearrangements in PUL organization are indicated by grey shapes or segments linking gene homologs. Conservation between homologous proteins is illustrated by identity percentages obtained by BLASTP alignments.

Common functions and influence of diet on the bovine rumen microbiome

Supplementary Figure 10. Non-metric multidimensional scaling (NMDS) gene counts by diet type (n= 77)

77 samples from four different diet groups: D= dairy cow (n=23), G= grazing diet (n=20), FH= fattening high starch + linseed diet (n=16), and FL= fattening low starch diet (n=18).

Supplementary Figure 11. Overlapping number of Genus, CAZy and KEGG Orthology (KO) across diets (n= 77)^1^

**Genus**

**CAZy**

**KO**

^1^77 samples from four different diet groups: D= dairy cow (n=23), G= grazing diet (n=20), FH= fattening high starch + linseed diet (n=16), and FL= fattening low starch diet (n=18).

Supplementary Figure 12. Size of the shared microbiome features among cattle fed four different diets

a


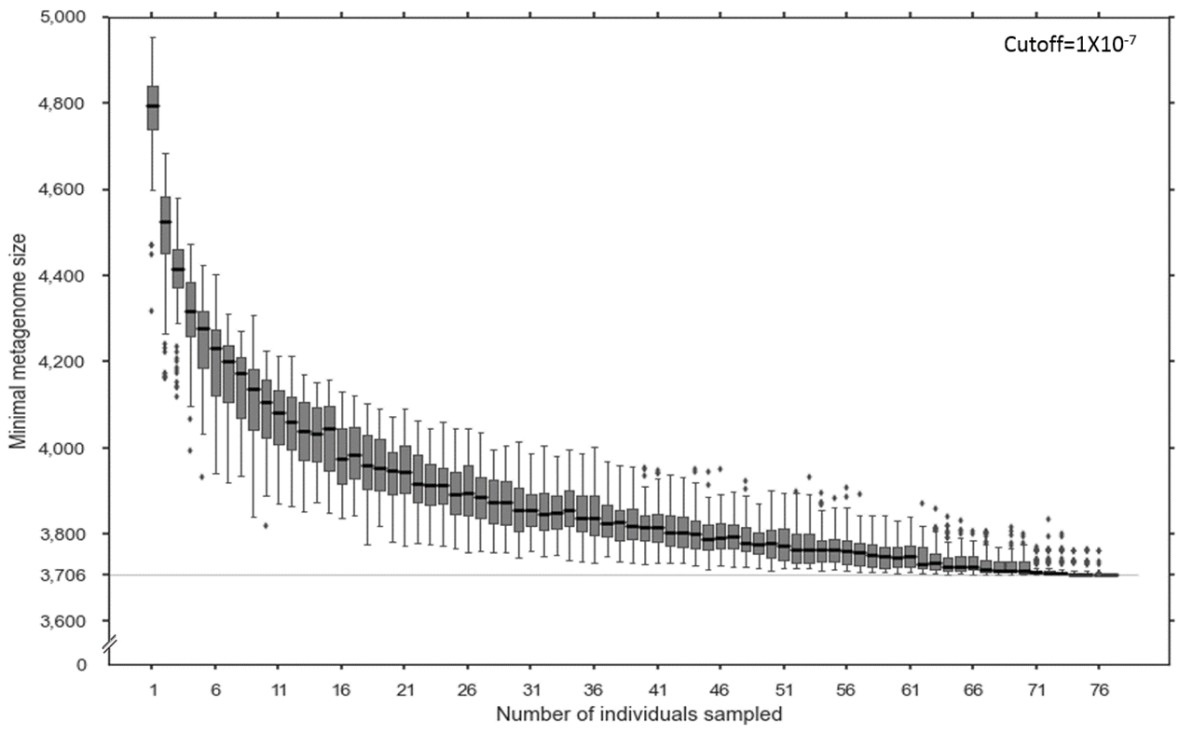


b


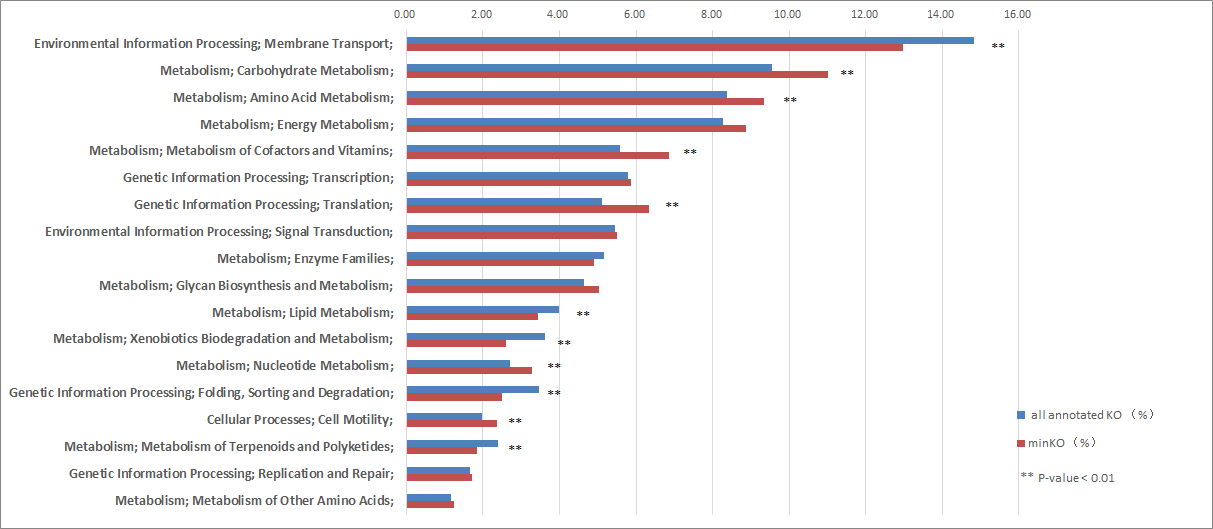


c

**
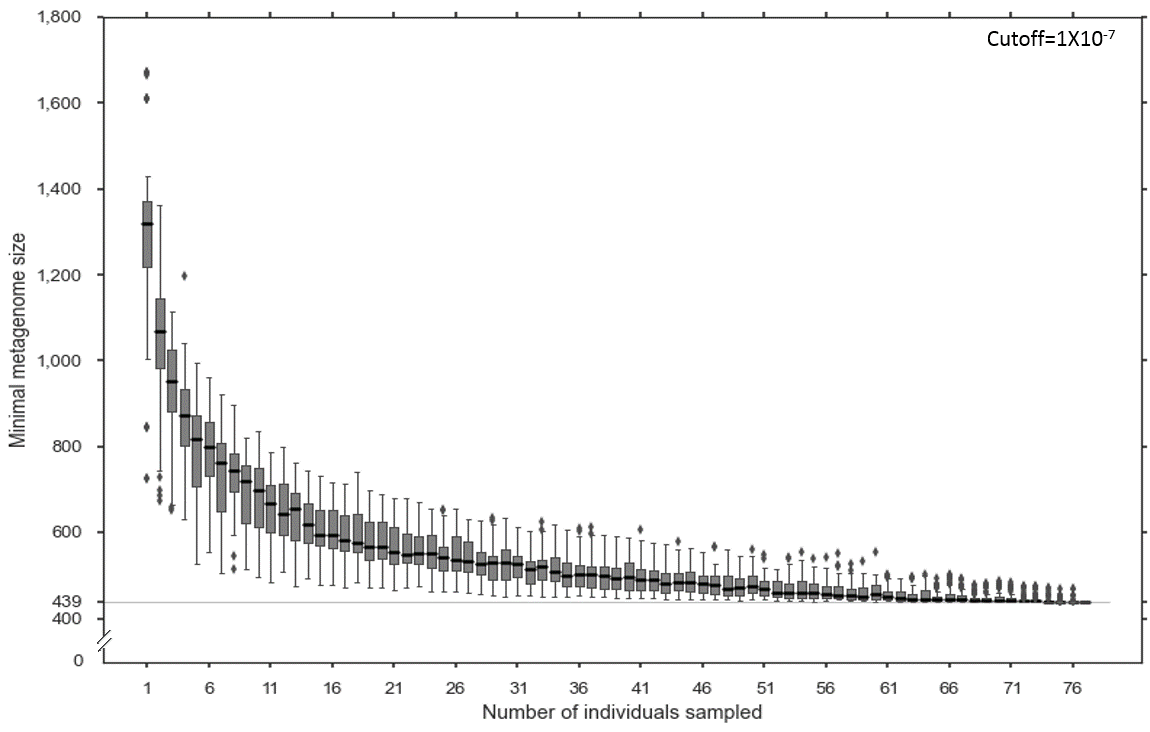
**

**d**

**
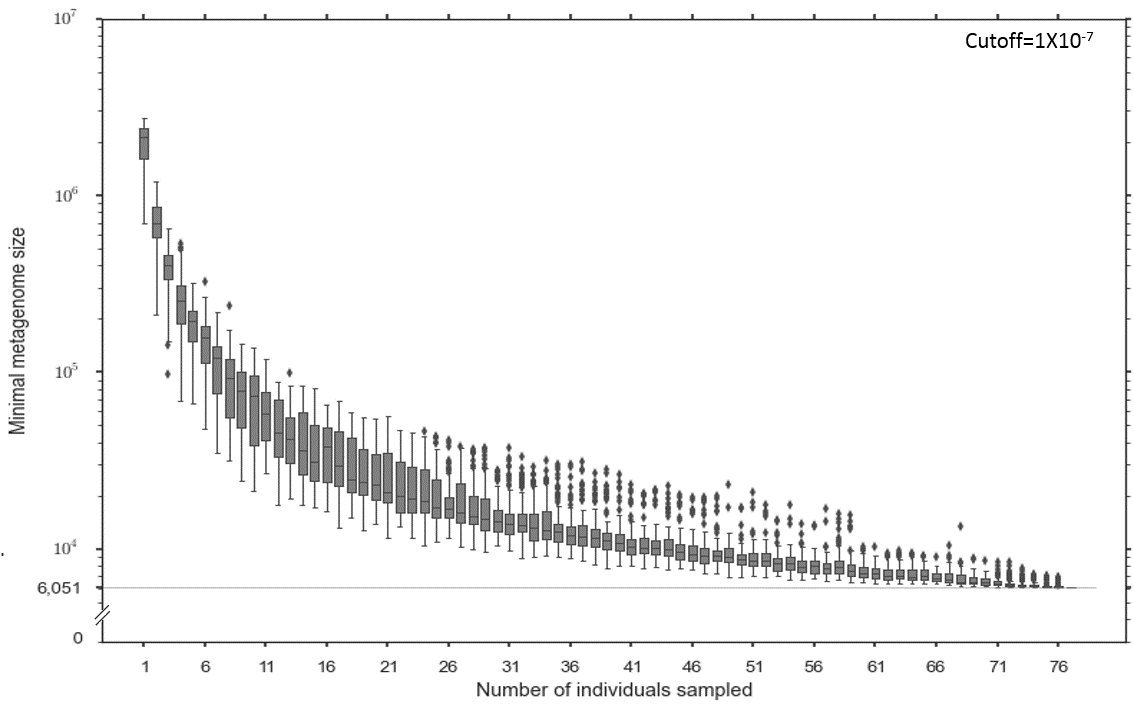
**

Supplementary Figure 12. Size of the shared microbiome features among cattle fed four different diets using different calculation methods than that of Figure 2 for comparison (see Methods section).

(a) The size of the minimal metagenome at KO levels (cutoff= 1X10^-7^). There are 5893 functionally annotated KO levels and a minimal set of 3706 functions was found for the 77 individuals sampled. (b) Change in the rate of all annotated KO and minimal KO in the second function level. (c) The size of the minimal metagenome at CAZy levels (cutoff= 1X10^-7^). There are 1974 functionally annotated CAZy levels and the minimal set found was of 439 functions. (d) The size of minimal metagenome at gene levels (cutoff= 1X10*^‑^*^7^), 6051 genes was the minimal set for the 77 individuals sampled.

Supplementary Figure 13. Cluster distribution by diet

Supplementary Figure 13 **(A)** Cluster distribution in Holstein and Charolais breeds based on CAZy families. For Holstein red label indicates dairy cow (D, n= 23) diet and green indicates grazing (G, n= 20) diet. For Charolais, dark blue indicates fattening high starch + linseed (FH, n= 16) diet and light blue indicates fattening low starch (FL, n=18) diet. Changes in differentially abundant families for each breed are shown in the tables below the graph (arrows indicate higher abundance). The complete list of families is available in Supplementary Table 10.

Supplementary Figure 13 **(B)** Cluster distribution in Holstein and Charolais breeds based on KEGG orthology. For Holstein red label indicates dairy cow (D) diet and green indicates grazing (G) diet. For Charolais, dark blue indicates fattening high starch + linseed (FH) diet and light blue indicates fattening low starch (FL) diet. Changes in the 10 most differentially abundant KO for each breed are shown below the graph. The complete list is available in Supplementary Table 11.

Supplementary Figure 13 **(C)** Cluster distribution in Holstein and Charolais breeds based on microbial genera. For Holstein red label indicates dairy cow (D) diet and green indicates grazing (G) diet. For Charolais, dark blue indicates fattening high starch + linseed (FH) diet and light blue indicates fattening low starch (FL) diet. Changes in differentially abundant genera are shown in the tables below the graph (arrows indicate higher abundance). The complete list is available in Supplementary Table 12.

**Holstein Charolais**


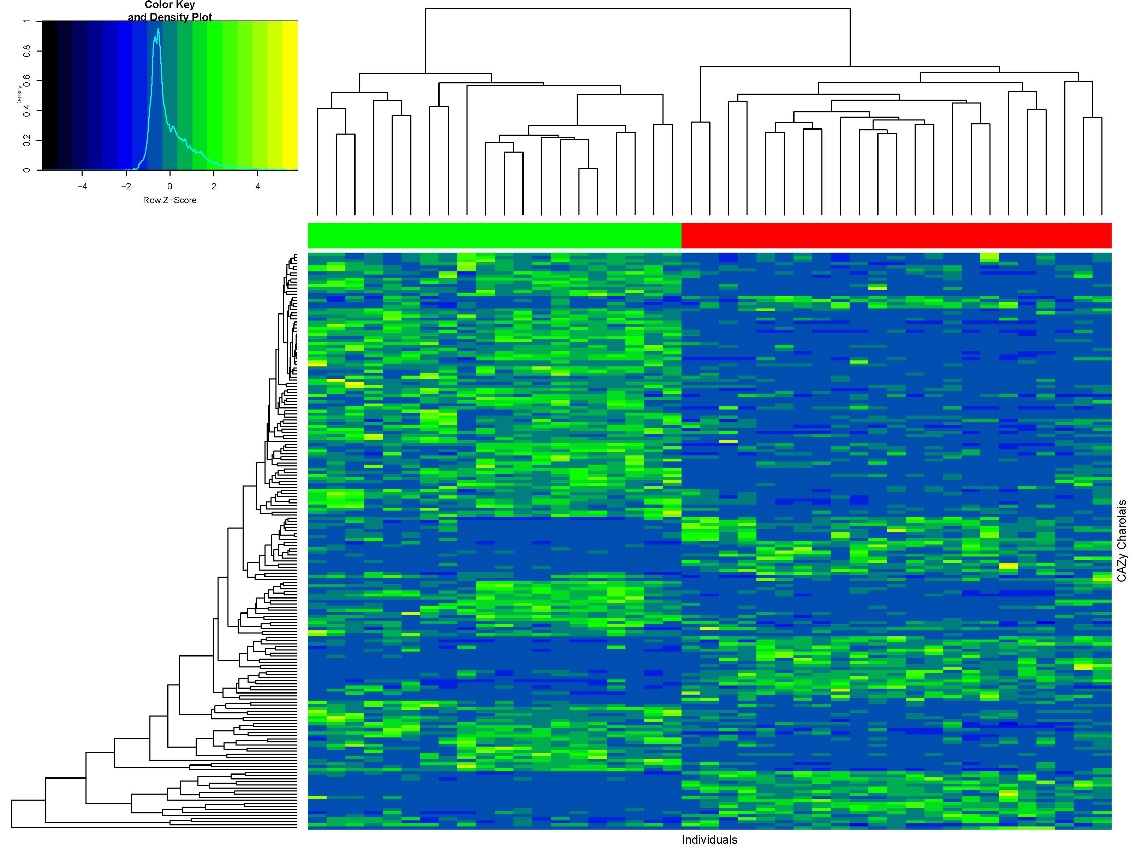

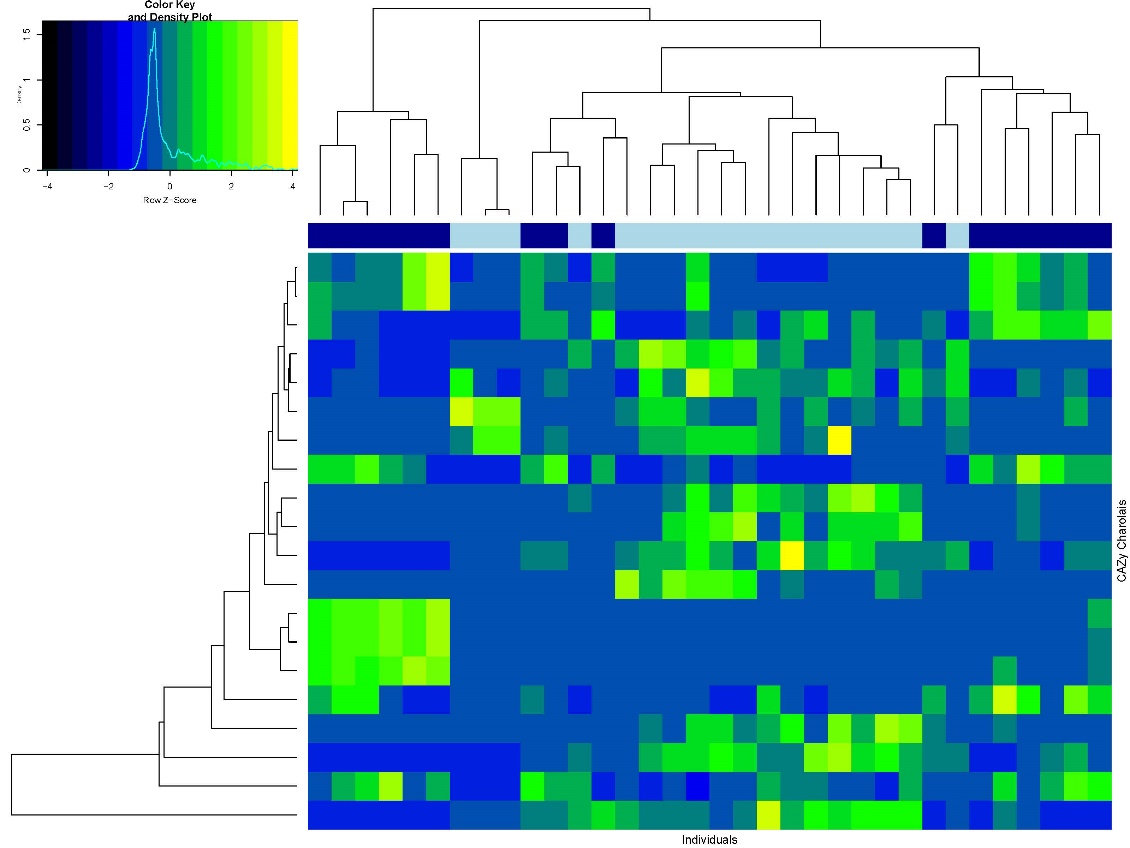


Supplementary Figure 13 **(D)** Cluster distribution in Holstein and Charolais breeds based on MAGs. For Holstein, red label indicates dairy cow (D) diet and green indicates grazing (G) diet. For Charolais, dark blue indicates fattening high starch + linseed (FH) diet and light blue indicates fattening low starch (FL) diet. The complete list is available in Supplementary Table 12.

Supplementary Figure 14. Effect of diet on the abundance of CAZy families in the bovine rumen metagenome.


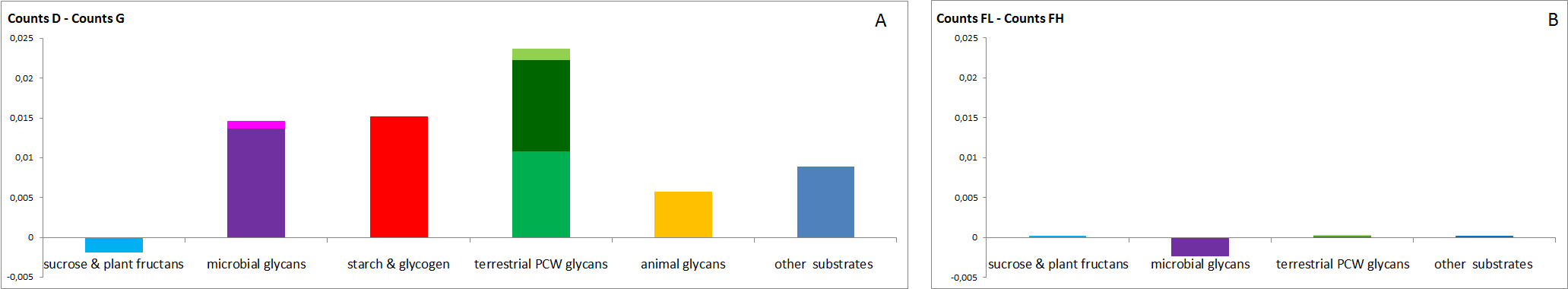


**Supplementary Figure 14.** Effect of diet on the abundance of CAZy families in the bovine rumen metagenome. Difference of abundance of CAZy families, clustered by substrate categories, between Dairy cow diet (D, n= 23) and grazing diet (G, n= 20) samples (A), and between fattening low-starch diet (FL, n= 16) and fattening high starch + linseed (FH, n= 18) samples (B). Only the most significant differentially abundant catabolic CAZy families (glycoside hydrolases, polysaccharide lyases, and family 35 of glycosyl transferases, of which the members are able to cleave osidic linkages of starch and glycogen by phosphorolysis) and their associated non-catalytic modules were taken into account (adjusted p value < 0.05). Color legend: pale purple, fungal glycans; dark purple, bacterial glycans; pale green, pectin; medium green, cellulose; dark green, hemicellulose. Polyspecific families containing members acting on various types of substrates are assigned to ‘other substrates’

Antibiotic resistance genes

Supplementary Figure 15. Antibiotic resistance genes

a)


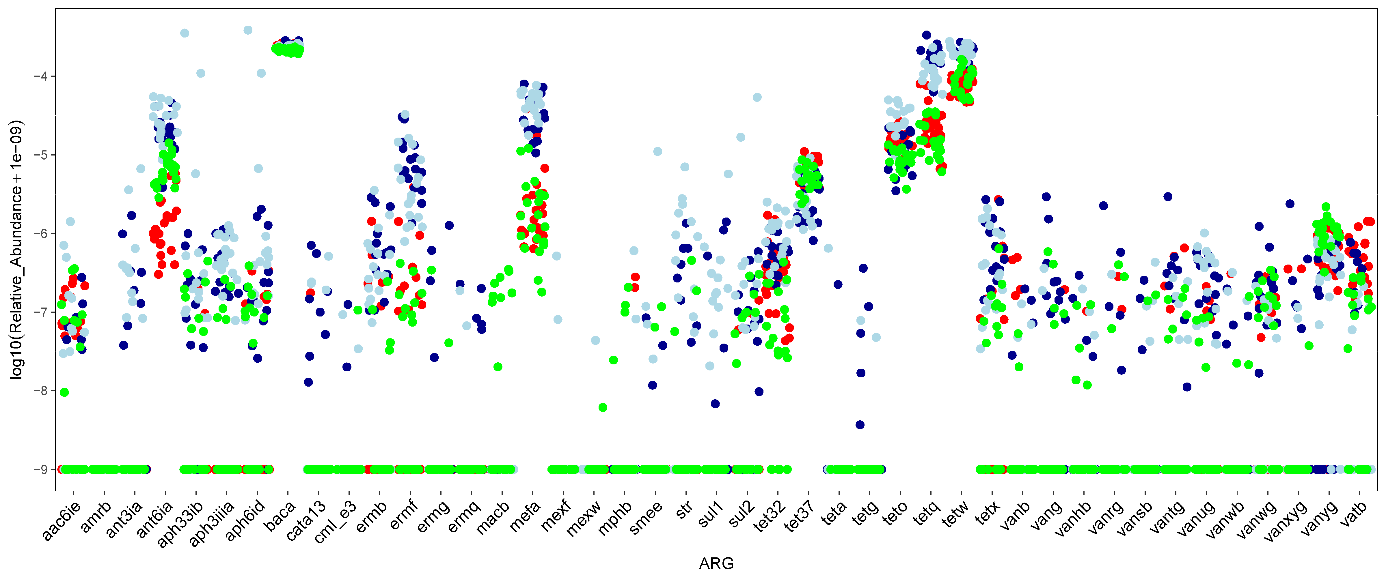

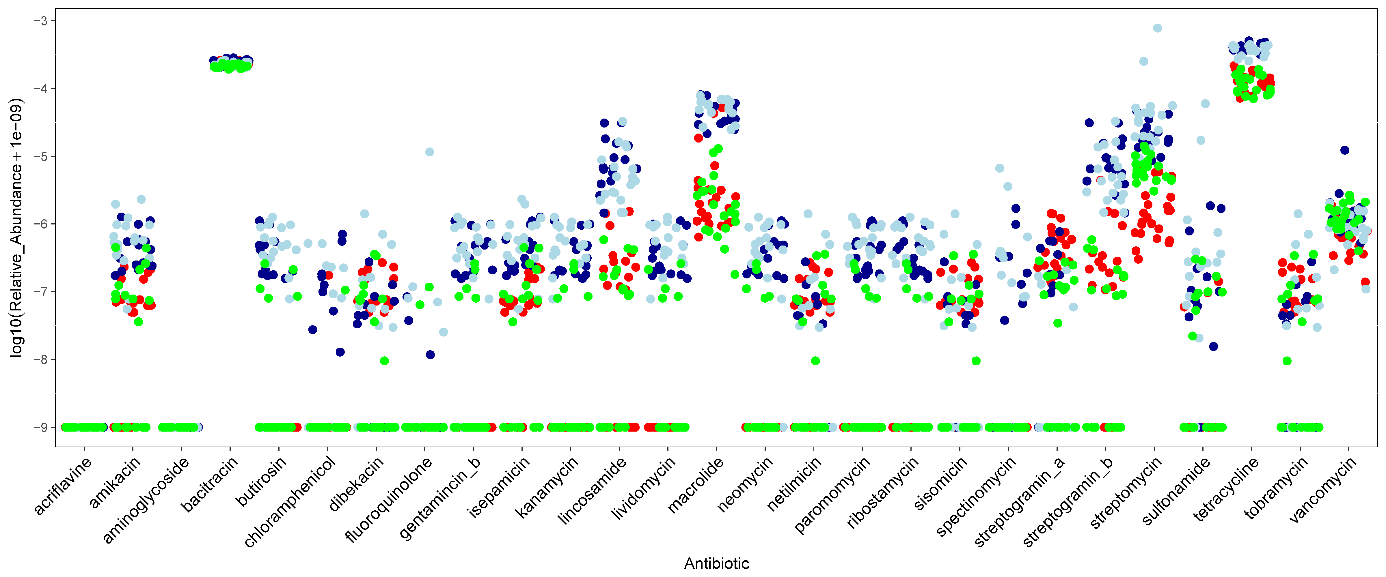

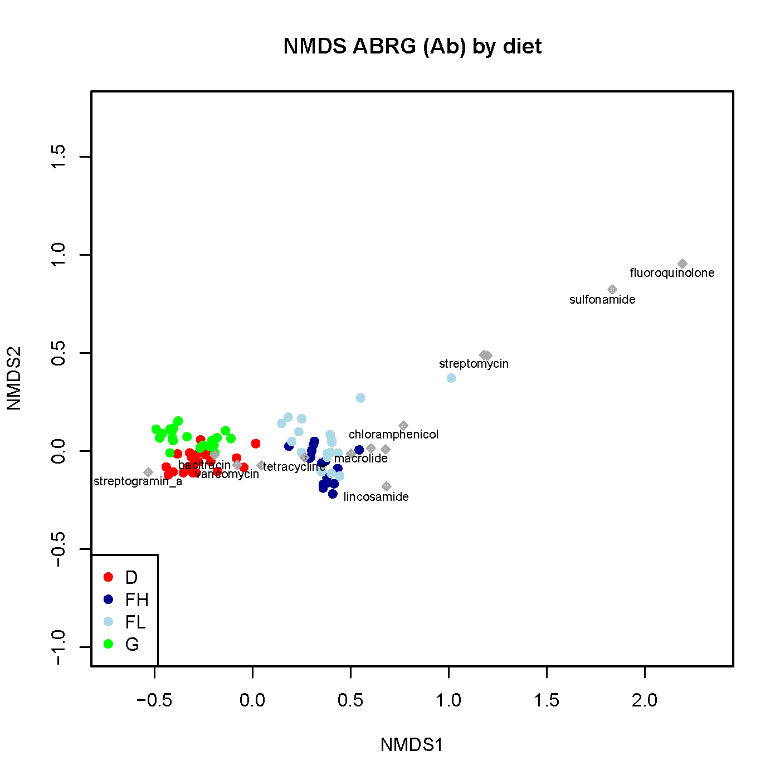


b)

Supplementary Figure 15. Prevalence of antibiotic resistance genes (ARGs) in the 77 samples cohort.

a) Relative abundances (log10 scale) of ARGs (top) and antibiotic types (bottom) found in each individual. In every column on the x axis each dot represents an animal, with colors according to diets: grazing (G; green), dairy (D; red), fattening high-start + linseed (FH; dark blue) and fattening low-starch (FL; light blue). The higher the vertical position of the dots on the y axis, the higher the relative abundance of the ARGs.

b) Non-metric multidimensional scaling (NMDS) biplot of the ARGs showing a separation between beef (FH and FL diets) and dairy breeds (D and G diets). Grey diamonds represent the individual ARG ordination onto the two-dimensional space, with the names of a subset of ARGs given in black.

## Methods

Construction of metagenome-assembled genomes (MAG) and taxonomic assignment

Supplementary Figure 16. Diagram describing the approach used for the construction of rumen metagenome-assembled genomes


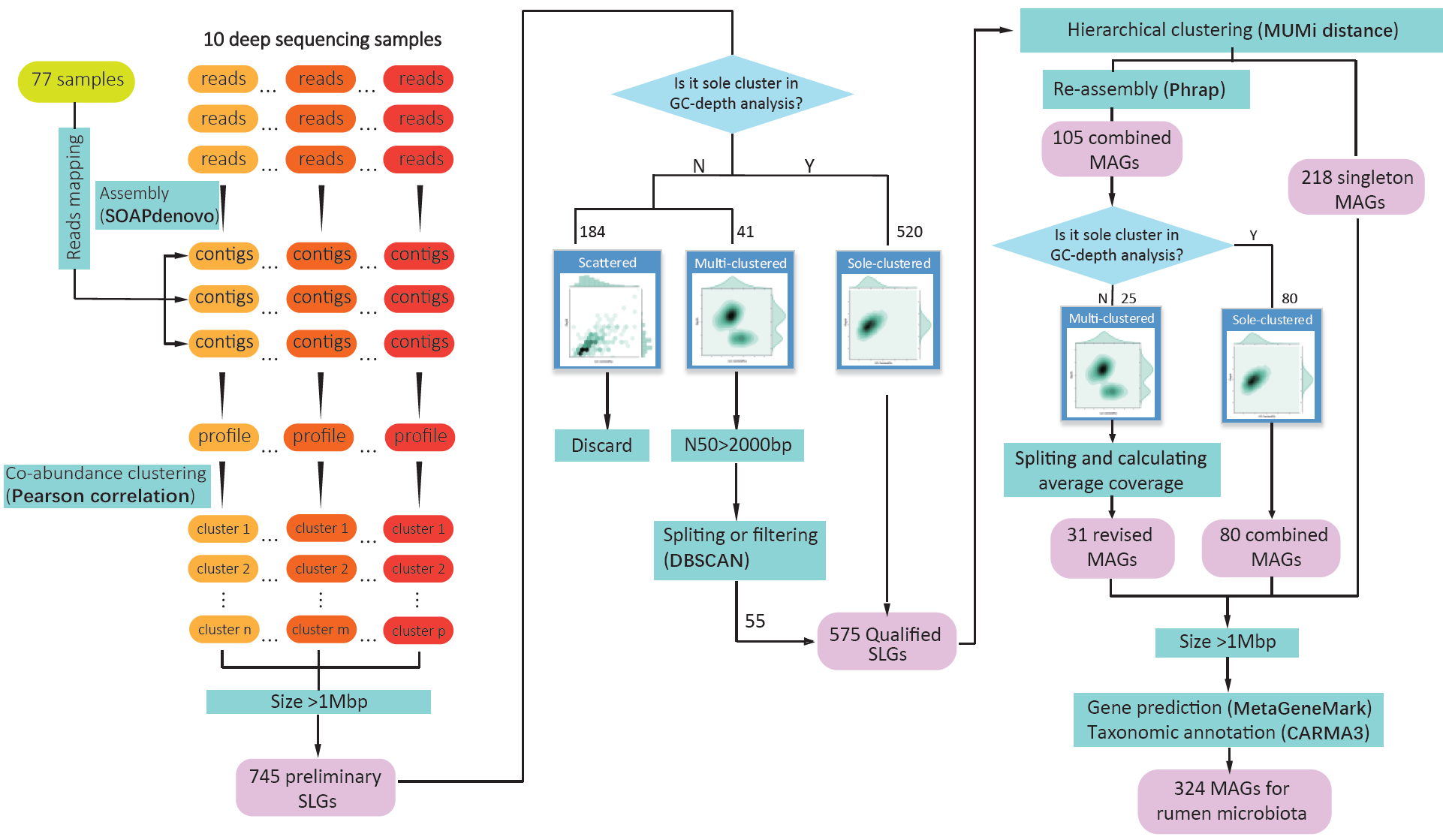


Supplementary Figure 16. Diagram describing the approach used for the construction of rumen metagenome-assembled genomes

Supplementary Figure 17. Percentage of 324 MAGs completeness and contamination.


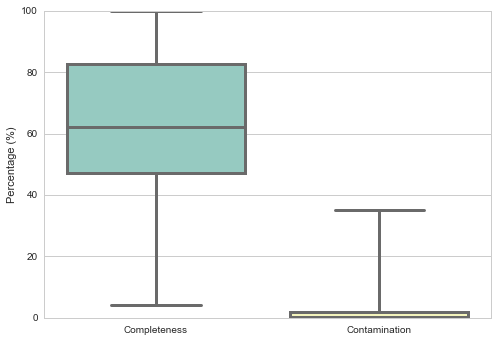

**Supplementary Figure 17. Percentage of 324 MAGs completeness and contamination.**

The median percentage of completeness and contamination reached 62.48% and 2.62%, respectively (see Supplementary Table 14) showing that the 324 MAGs have high completeness and low contamination.

Supplementary Figure 18. Completeness, Contamination and Strain heterogeneity in MAGs.

**
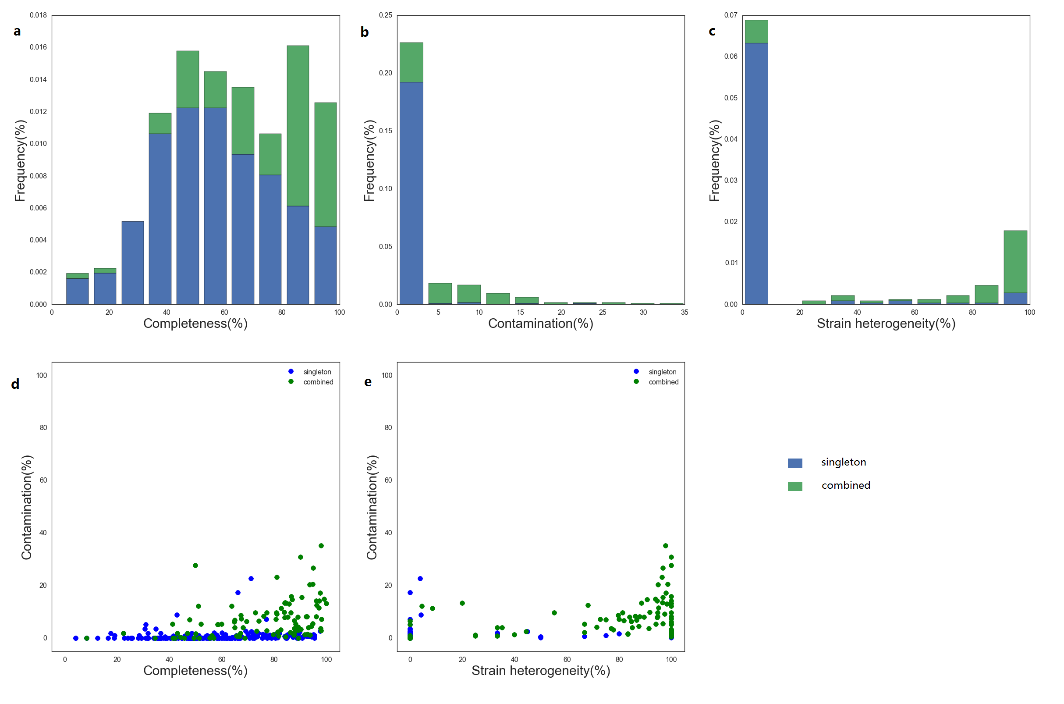
**

Supplementary Figure 18. Completeness, Contamination and Strain heterogeneity in singleton (blue) and combined (green) MAGs.

(a)Proportion of completeness between combined and singleton MAGs. Most MAGs show a completeness greater than 40%. (b) Frequency of contamination between combined and singleton MAGs. Contamination in most MAG is 0, the rest of MAGs contamination is mainly due to combined MAGs. (c) Proportion of strain heterogeneity between combined and singleton MAGs. Strain heterogeneity in most singleton MAG is 0, whereas most combined MAGs have a strain heterogeneity greater than 70%. (d) When MAG completeness is greater than 40% then most of MAGs contamination is less than 10%. (e) When the MAGs contamination greater than 10%, the contamination of MAGs (mostly combined MAG) basically come from strain heterogeneity.

Quality assessment and taxonomic annotation of MAGs

Supplementary Figure 19. The distribution of MAG from the bovine rumen and pig and mice feces metagenomes at different taxonomic levels.

a)

b)

c)

Supplementary Figure 19. The distribution of MAG from the bovine rumen and pig and mice feces metagenomes at different taxonomic levels (a). MAG distribution at phylum (b) and genus (c) level.

Supplementary Figure 20. Relationship between coverage and depth for MAG


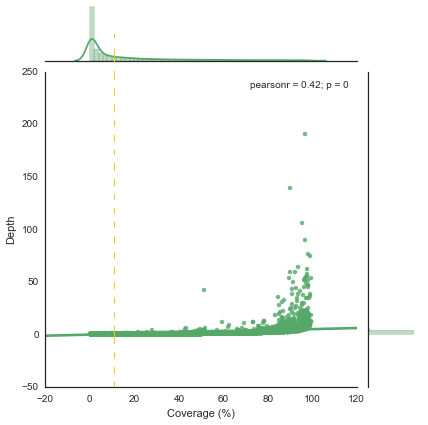


Supplementary Figure 20. Relationship between coverage and depth for MAG. Each point represents one MAG in one of the 77 samples. Histogram in the top and right-hand indicates the fraction of coverage and depth, respectively. When the coverage is less than 10%, then the value of depth is close to 0.

Supplementary Table 1. Summary information of the bovine rumen prokaryotic gene catalog described in this study

Supplementary Table 1. Summary information of the bovine rumen prokaryotic gene catalog described in this study

| Total number of genes | Number of complete genes | Total length (bp) | Average length (bp) | N50 (bp) | N90 (bp) | Max length | Min length | Taxonomic Annotation (Phylum Level) | | Taxonomic Annotation  (Genus Level) | | KEGG Annotation | | Cazy Annotation | |  |
| --- | --- | --- | --- | --- | --- | --- | --- | --- | --- | --- | --- | --- | --- | --- | --- | --- |
| 13,825,880 | 5,410,738 | 9,896,715,705 | 715.81 | 906 | 396 | 40,812 | 102 | | 5,899,724 (42.67%) | | 1,205,758 (8.72%) | | 5,947,496 (43.02%) | | 174,226 (3.22%) | |

Supplementary Table 2. Summary information of the bovine rumen gene catalog described by Hess et al. (2011) [[1](#_ENREF_1)] pre- and post-filtering

Supplementary Table 2. Summary information of the bovine rumen gene catalog described by Hess et al. (2011) [[1](#_ENREF_1)] pre- and post-filtering

|  | Number of genes | Number of bases | Average length | Max length | Min length | N50 (bp) | N90 (bp) |
| --- | --- | --- | --- | --- | --- | --- | --- |
| Published data | 2,547,270 | 1,379,576,700 | 541.59 | 319,74 | 60 | 744 | 261 |
| Filtered data | 2,461,518 | 1,218,405,383 | 494.98 | 319,74 | 100 | 681 | 231 |

Supplementary Table 3. Excel file “Suppl_Table_3_comparison_genomes”

**Supplementary Table 3**. Whole-genome similarity between MAGs from this work, Hungate1000 genomes [[5](#_ENREF_5)] and MAGs from Stewart et al. [[3](#_ENREF_3), [4](#_ENREF_4)].

Supplementary Table 4. Excel file “Suppl_Table_4_10+77samples”

**Supplementary Table 4.** Metagenomic data production from bovine rumen samples

Supplementary Table 5. Excel file “Suppl_Table_5_catalogs_rumen_pig_mouse”

**Supplementary Table 5.** Number of genes and taxonomic annotation information for bovine rumen and human, pig and mouse feces gene catalogs

Supplementary Table 6. Detailed annotation information at genus level for rumen, pig and mice catalogs

Table 6. Detailed annotation information at genus level for rumen, pig and mice catalogs

| **Genus** | **Rumen** | **Pig** | **Mice** |
| --- | --- | --- | --- |
| *Prevotella* | 39.22% | 24.85% | 1.79% |
| *Treponema* | 13.07% | 1.78% | 0.01% |
| *Butyrivibrio* | 10.44% | 3.82% | 5.83% |
| *Methanobrevibacter* | 9.62% | 1.83% | 0.00% |
| *Ruminococcus* | 8.80% | 9.40% | 2.45% |
| *Bacteroides* | 4.65% | 10.44% | 25.01% |
| *Clostridium* | 3.26% | 10.27% | 27.08% |
| *Fibrobacter* | 2.08% | 0.12% | 0.00% |
| *Eubacterium* | 1.56% | 6.67% | 2.66% |
| *Roseburia* | 0.77% | 2.51% | 2.83% |

Supplementary Table 7. Excel file “Supplementary Table 7_CAZy-counts_human_rumen_mouse_pig_catalogs”

**Supplementary Table 7.** Proportion of carbohydrate active enzymes (CAZy) in the rumen microbiome of cattle and gut microbiomes of human, pig and mouse.

Supplementary Table 8. Excel file “Supplementary Table 8_DA_Fibrobacter_CAZy”

**Supplementary Table 8.** Effect of diet on the abundance of CAZy genes assigned to *Fibrobacter succinogenes* in the bovine rumen metagenome. Gene counts of the differentially abundant genes in the D and G samples (spreadsheet ‘Holstein’), and in the FL and fattening high starch + linseed diet (FH) samples (spreadsheet ‘Charolais’). Similarity of 1262 CAZy Fibrobacteres genes enumerated in the catalogue to *Fibrobacter succinogenes* S85.

Supplementary Table 9. Differential abundance analysis

Supplementary Table 9. Number of differentially abundant Genus, MAG, CAZy and KO within each breed detected by differential abundant analysis.

| **Breed** | **Genus** | **MAG** | **CAZy** | **KO** |
| --- | --- | --- | --- | --- |
| Holstein | 106 | 188 | 146 | 2445 |
| Charolais | 44 | 20 | 8 | 208 |

Supplementary Table 10. Excel file “Suppl_Table_10_DA_CAZy”

**Supplementary Table 10.** Effect of diet on the abundance of CAZy families in the bovine rumen metagenome. Gene counts of the differentially abundant families of glycoside hydrolases, polysaccharide lyases, family 35 of glycosyl transferases, carbohydrate-binding modules, dockerins and cohesins in the dairy cow (D) and grazing (G) diets samples (sheet ‘Holstein’), and in the fattening low-starch diet (FL) and fattening high starch + linseed diet (FH) samples (sheet ‘Charolais’). The targeted substrates are indicated for each family.

Supplementary Table 11. Excel file “Suppl_Table_11_DA_KO”

**Supplementary Table.** Effect of diet on the abundance of KO in the bovine rumen metagenome

Supplementary Table 12. Excel file “Supplementary Table 12_DA_Genus_MAG”

**Supplementary Table 12.** Effect of diet on the abundance of genera and MAGs in the bovine rumen metagenome.

Supplementary Table 13. Vector fitting Table

Pheno: phenotype; LW: live weight; VFA: volatile fatty acid; C2: acetate; C3: propionate, iC4: iso-butryrate; C4: butyrate; iC5: iso-valerate; C5: valerate; C6: caproate; C2_C3: acetate to propionate ratio; iso VFA: sum of iC4 and iC5; minorVFA: sum of iso acids, valerate and caproate.

See [[6](#_ENREF_6)] for metadata information.

Supplementary Table 14. Excel file “Supplementary Table 14_Assessment of 324_MAG”

**Supplementary Table 14.** Evaluation of assembly quality for 324 rumen MAGs

Supplementary Table 15. Excel file “Supplementary Table 15_rumen-pig-mice_MAG_annotation”

**Supplementary Table 15.** Taxonomic annotation for MAGs catalog of rumen, pig and mice by CARMA3

Supplementary Table 16. Excel file “Suppl_Table_16_Hungate_genomes”

**Supplementary Table 16.**  List of microbial genomes from the Hungate1000 project used in this work

**References for Figures and Tables captions**

1. Hess M, Sczyrba A, Egan R, Kim TW, Chokhawala H, Schroth G, et al. Metagenomic discovery of biomass-degrading genes and genomes from cow rumen. Science. 2011;331 6016:463-7. doi:10.1126/science.1200387.

2. Wallace R, Rooke J, McKain N, Duthie C-A, Hyslop J, Ross D, et al. The rumen microbial metagenome associated with high methane production in cattle. BMC Genomics. 2015;16 1:839. doi:10.1186/s12864-015-2032-0.

3. Stewart RD, Auffret MD, Warr A, Walker AW, Roehe R and Watson M. Compendium of 4,941 rumen metagenome-assembled genomes for rumen microbiome biology and enzyme discovery. Nat Biotech. 2019;37 8:953-61. doi:10.1038/s41587-019-0202-3.

4. Stewart RD, Auffret MD, Warr A, Wiser AH, Press MO, Langford KW, et al. Assembly of 913 microbial genomes from metagenomic sequencing of the cow rumen. Nat Commun. 2018;9 1:870. doi:10.1038/s41467-018-03317-6.

5. Seshadri R, Leahy SC, Attwood GT, Teh KH, Lambie SC, Cookson AL, et al. Cultivation and sequencing of rumen microbiome members from the Hungate1000 Collection. Nat Biotech. 2018;36 4:359-67. doi:10.1038/nbt.4110.

6. Li J, Zhong H, Ramayo-Caldas Y, Terrapon N, Lombard V, Potocki-Veronese G, et al. Supporting data for "A catalog of microbial genes from the bovine rumen unveils a specialized and diverse biomass-degrading environment". GigaScience Database. 2018. doi:10.5524/100391.
