# Supplementary material for: A catalog of microbial genes from the bovine rumen unveils a specialized and diverse biomass-degrading environment
Source: Gigascience. 2020 May 30;9(6):giaa057. doi: 10.1093/gigascience/giaa057 (PMC7260996; doi:10.1093/gigascience/giaa057)
Supplement: giaa057_Response_to_Reviewer_Comments_Original_Submission [file giaa057_response_to_reviewer_comments_original_submission.pdf]

Author's Response To Reviewer Comments

Close

I wish to highlight two main concerns of reviewer 1 that need to be carefully addressed before we can make a decision on acceptance:

1) The reviewer points out that you used SOAPdenovo v1, which is not suitable for this type of data, according to the reviewer (and also meanwhile replaced by a newer version). I feel the most clean way to address this concern would be to redo the analysis with more appropriate software.

2) I also agree with reviewer 1 that more extensive comparisons with existing datasets are essential before we can make a decision on acceptance.

Please refer to the detailed comments of both reviewers below.

If you are able to fully address these points, we would encourage you to submit a revised manuscript to GigaScience. Once you have made the necessary corrections, please submit online at:

https://www.editorialmanager.com/giga/

If you have forgotten your username or password please use the "Send Login Details" link to get your login information. For security reasons, your password will be reset.

Please include a point-by-point within the 'Response to Reviewers' box in the submission system. Please ensure you describe additional experiments that were carried out and include a detailed rebuttal of any criticisms or requested revisions that you disagreed with. Please also ensure that your revised manuscript conforms to the journal style, which can be found in the Instructions for Authors on the journal homepage.

Apologies again that it took us so long to get the two reports - as I said previously, one of the agreed reviewers did let us down and never returned a report, but thankfully I found another expert (reviewer 2 below) who could step in.

The due date for submitting the revised version of your article is 25 Oct 2019.

I look forward to receiving your revised manuscript soon.

Best wishes,

Hans Zauner
GigaScience
www.gigasciencejournal.com

Dear editor,
We are grateful for giving us the opportunity to revise the manuscript. All modifications made in the revised manuscript are highlighted in yellow to facilitate reading. Below is the point by point reply to reviewers' comments.
The reason to use SOAPDenovo is because this was a long-term project and when the analysis started the newer tools mentioned by the reviewer were not available. For the concern about the appropriateness of the assembler we performed a comparison between SOAPDenovo and MEGAHIT using a reduced dataset. Please see the response to the reviewer for detailed information. Instead of redoing all the analysis, we first performed a comparison to effectively assess whether this older tool could lead to false gene predictions. This comparison showed that genes identified by SOAPDenovo were comparable to MEGAHIT although the latter produced a higher number of genes. Based on this

information we considered that the data produced by the original pipeline cannot be questioned and decided not to modify the method. Also taking into consideration all the implications that this would have had on other aspects of the work such as the comparison to other catalogs and the analysis of CAZy.
For the second concerns that you highlighted in your message we included in the revised manuscript the comparison with the MAGs dataset suggested by the reviewer (although at the time the dataset that was originated from the reviewer's lab was not peer reviewed).
We hope that the changes made to the revised manuscript and replies to reviewers' comments are satisfactory and the manuscript can be published in GigaScience.

Kind regards,

Diego Morgavi
On behalf of all authors

Response to reviewers' comments

Reviewer reports:

Reviewer #1: The authors present a collection of microbial genes from the bovine rumen. Accurate gene catalogs are an important resource for an environment that is largely underrepresented in databases and the rumen is a particularly interesting environment. They also present data on differences in microbiome composition, abundance of different species and microbial gene functions between cows from different genetic backgrounds fed on different diets. While the paper is interesting, and the resources produced are likely important I have some concerns.
Authors'_reply: Thank you for your very careful and constructive review of our paper, and for the comments, corrections and suggestions that ensued. This has resulted in major modification of the revised paper. Please see below for the specific responses to comments.

The authors use SOAPdenovo v1.06 for their assemblies. I question the appropriateness of this choice given SOAPdenovo's documentation recommends MEGAHIT, a tool designed to handle metagenomic data which SOAPdenovo is not designed for.
The paper for MEGAHIT, by the same authors as SOAPdenovo, also states "Note that SOAPdenovo2 and Minia are designed to assemble a single genome. For metagenomic data, which involve numerous different genomes with uneven depth coverage and cross-genome repeats, specifically designed algorithms are required to achieve good assembly quality." There has also been a more recent version of SOAPdenovo than the one used, SOAPdenovo2. Additionally, other tools specifically designed for metagenomic assembly have been available for a number of years, e.g. IDBA-UD. It would have been more appropriate to use a tool designed for metagenomic assembly. My concern is that older tools don't tend to perform as well, and a tool designed to work on a single genome may have produced false joins in the contigs which in turn could lead to some false gene predictions and truncations. What is the justification for using this tool?

Authors'_reply: This is a valid comment as improved performance is expected from newer tools. We used SOAPdenovo because the initial analysis of the dataset started before the MEGAHIT assembler was available. To address the reviewer's concerns, we compared the assembly of a data subset using SOAPdenovo v1.06 and the latest version of MEGAHIT and found that the accuracy of gene assembled by SOAPdenovo was comparable to that of MEGAHIT, although the latter produced more genes (see below). As for the use of the SOAPdenovo version, SOAPdenovo2 incorporates SOAPdenovo v1.05 and v1.06 as integral assembly components and thus SOAPdenovo v1.06 showed performance close to SOAPdenovo2 as described in [Comparing and Evaluating Metagenome Assembly Tools from a Microbiologist's Perspective - Not Only Size Matters; 10.1371/journal.pone.0169662].
We compared assembly results based on SOAPdenovo v1.06 (our results) to those based on MEGAHIT v2.19 using An.552 (one of the ten deep sequenced samples, Charolais, bull, 350PE, 122.1G). Following assembly we performed a CD-HIT analysis to identify similar (redundant) genes between the two gene sets, and to evaluate to what extent the assembled genes by SOAPdenovo v1.06 could be represented by those genes assembled by MEGAHIT v2.19 and vice versa. The parameters (n 8 -d 0 -g 1 -T 6 -G 0 -aS 0.9 -c 0.95) were the same as we used for establishing a non-redundant rumen gene catalog. Indeed, as shown in rebuttal Table 1, MEGAHIT v2.19 showed better performance (longer contigs and more genes) than SOAPdenovo v1.06 (Rebuttal Table 1). Notwithstanding, regarding the main concern of the reviewer about false assignment of genes, the comparison of the CD-HIT shows that 853,085

genes (50.13%) assembled by SOAPdenovo v1.06 were non-redundant genes and 737,617 of these genes were also assembled by MEGAHIT v2.19. Most importantly, 842,886 genes assembled by SOAPdenovo v1.06 were also present in the MEGAHIT v2.19 dataset (Rebuttal Table 2). Thus, 92.88% of genes [(737,617 + 842,886) / 1,701,641] assembled by our original SOAPdenovo pipeline were identified by the MEGAHIT pipeline (showing ≥ 95% identity and ≥90% coverage).
These data indicate the high accuracy of most genes assembled by SOAPdenovo, though it has generated a lesser number of genes than a newer tool such as MEGAHIT.
Contigs Sample An.552 SOAPdenovo v1.06 MEGAHIT v2.19
Total size(Mbp) 1337.53 1921.51
# of Contigs 830,535 1034,236
# of Contigs >1k bp 356,354 464,849
Average length(bp) 1610 1858
N50(bp) 2226 3052
Longest contig(bp) 364889 508659
Genes Sample An.552 SOAPdenovo v1.06 MEGAHIT v2.19
Total size(Mbp) 1218.94 1747.90
# of genes 1701,641 2429,342
# of genes >1k bp 347,376 521,905
Average length(bp) 716.33 719.50
Longest(bp) 29,580 38,499
Rebuttal Table 1 Summary of assembly results from SOAPdenovo and MEGAHIT

MEGAHIT v2.19 SOAPdenovo v1.06 # of non-redundant genes for An.522
# of genes 2,429,342 1,701,641
# of representative genes 1,599,864 853,085 2,452,946
# of redundant genes 829,478 848,556 NA
# of redundant genes represented by genes assembled using SOAPdenovo 737,617 5,670 NA
# of redundant genes represented by genes assembled using MEGAHIT 91,861 842,886 NA
Rebuttal Table 2 Summary of CD-HIT results from SOAPdenovo and MEGAHIT

The authors compare their results to 2 other publicly available datasets, Stewart et al., 2018 and Hess et al., 2011 and demonstrate novelty in their data compared to the other two. However, beyond mapping rates the method of comparison (e.g. for %IDY) is not clear, nor is it clear what tool was used for mapping. Please add a section to the methods specifically describing methods for comparisons with other datasets and specify in results sections which analysis lead to the result.

Authors'_reply: We added this information on the methods section under the subheading "Comparisons between 324 MAGs and public rumen microbial genomes."
The added paragraph reads: "High-quality reads of 77 rumen samples were aligned against the assemblies of the 324 MAGs in current study, of the nearly 5,000 MAGs from Scottish cattle [6, 7], of the 409 genomes of microbes isolated from rumen (Hungate 1000; Supplementary Table 17) [5], and of the 15 MAGs from JGI using SOAP2 (≥ 95% identity) [46]. Mapping ratios of 77 rumen samples to the rumen microbial genome collections from the above studies were calculated as number of mapped reads to number of total reads. Whole-genome similarities between current 324 MAGs and published rumen microbial genomes were calculated using MUMmer. MAGs showing MUMi values less than 0.54, a suggested threshold for generating a species level MAG [53], with published rumen microbial genomes were considered as novel MAGs (Supplementary Table 3)."

Stewart et al. have recently produced a much more extensive dataset (https://www.biorxiv.org/content/10.1101/489443v1, data available: https://datashare.is.ed.ac.uk/handle/10283/3224) producing almost 5000 MAGs. Additionally, there are other rumen datasets available which should be included to give a complete picture of the novelty in the authors' dataset and give some idea of the extent of novelty still unexplored in the rumen, e.g. Parks et al., Solden et al. and Svarstrom et al.

Authors'_reply: Thanks for the constructive suggestion.
As indicated in our response above, in the revised manuscript we further compared the whole-genome similarities between the current 324 MAGs and this latest published rumen microbial genomes (4,907 MAGs, https://datashare.is.ed.ac.uk/handle/10283/3224 [please note that 36 MAGs out of the 4,941 MAGs described by Stewart et al. 2019, were not available for downloading when the site was accessed

in August 2019]) using MUMmer.
As shown in the revised Supplementary Table 3, 135 MAGs from the current study displayed MUMi values less than 0.54, which is a suggested threshold for generating a species level MAG [53], with 4,907 published rumen microbial genomes, and 123 MAGs displayed MUMi values less than 0.54 with the three most comprehensive rumen microbial genome datasets. These data suggest that our MAGs are valuable dataset to provide novel information on rumen microbiome.

The comparisons carried out by the authors focus on similarities between MAGs and similarities between CAZymes, however the comparison between the full set of proteins the authors identified, and previously identified proteins is minimal. The authors have used CD-HIT once on their own data to remove redundant genes and once with a set of Hess et al.'s genes, but I suggest the authors collapse the protein set following UniRef guidelines at 100%, 90% and 50% similarity using their own proteins, and again using their own proteins and all other known rumen proteins to fully demonstrate novelty and reduce redundancy. This should be done at least for Hungate 1000 and the largest dataset of Stuart et al., but would preferably include other datasets.
There is mention that this was partly done for the Hess et al. dataset, however the methods described are not detailed or reproducible"13.83 M genes from current study and 2.46 M genes from JGI were pooled together to identify shared genes using CD-HIT".
Recommended CD-HIT parameters:
ID=100%: -c 1.0
ID=90%: -c 0.90 -n 5 -s 0.80
ID=50%: -c 0.50 -n 3 -s 0.80
(using output of previous level as input of lower level)

Following this the authors can report the number of clusters in their dataset that are novel relative to the other datasets.

Authors'_reply: Thanks for this comment.
We hereby have revised our methods with details of parameters. For instance, the above sentence was revised as "13.83 M genes from current study and 2.46 M genes from JGI were pooled together to identify shared genes using CD-HIT with ≥95% identity and ≥90% overlap [44]." The parameters for CD-HIT we used in this study were: -n 8 -d 0 -g 1 -T 6 -G 0 -aS 0.9 -c 0.95.
We showed that the whole-genome sequence similarities between 123 MAGs of our study and all collected rumen microbial genomes of the three other large studies were less than 0.54 (Revised Supplementary Table 3). Only 24 MAGs showed species-level or higher whole-genome sequence similarities (≥0.54) with rumen isolates of the Hungate 1000 (Revised Supplementary Table 3).

Additionally, we also reported that the reads mapping ratios of 77 samples to the 13.8M rumen gene catalog, ranged from 32 to 45% in the four diet groups (Supplementary Figure 1), which were higher than that of the latest rumen microbial genome set (n=4,907, 20% to 40%,revised Figure 1), and that of the Hungate 1000 dataset (<10%, revised Figure 1). Together, these sequence-based analyses indicate that there is novelty in our datasets and that there is low overlapping with other datasets.

The authors identify CAZymes by comparing predicted proteins to the CAZy database and to Hidden Markov models built from each CAZy family. Ideally, a tool such as dbCAN2 should be used to annotate CAZymes. This tool is automated and uses multiple methods to annotate CAZymes and is generally more accurate than using one method. Ideally all genes should be annotated with KEGG (which was used) and Uniref (which was not).

Authors'_reply: CAZyme annotation was realized by Bernard Henrissat and his team, i.e. the research scientists who have created, maintain and update the family classification of CAZymes that is the original classification from which dbCAN is based from. CAZyme annotation by Henrissat and his team relies on semi-manual annotation, i.e. it is automated for high-similarity levels proteins but is manually curated for the twilight zone (mid-to-low similarity levels) where homology cannot be distinguished automatically from noise.
Fully-automated methods such as dbCAN, do not reach the same high-quality as they notably apply a unique threshold for all families, and use profiles that are sometimes so considerably degenerated that they retrieve many false positives. Inaccuracies in dbCAN have been highlighted by others [Barrett and Lange, Biotech Biofuels, 2019, 12:102] who have reported important failures of dbCAN. In consequence we prefer to use annotations made by those who have almost 30 years' experience in CAZyme science and curation rather than unsupervised "push button" tools.

Parts of the methods section are a little difficult to follow, for example the first mention of scaftigs is on page 23 and it is not immediately clear these have come from the assembly on page 21. Suggest the title of section on page 21 include mention of scaftigs (i.e. Construction of the rumen microbial scaftigs and gene catalog) and/or including the details of the assembly tool again in the section on page 23. The authors should review the methods and ensure that it is clear where the data used for each section originated. Additionally the authors stated multiple times that methods were "as described previously" or similar, it would be helpful to have more of a description so there is less need to go searching for the methods and more ease of reproducibility, even if this was just added to the supplement. Additionally, in some cases the papers used describe specific parameters or describe manual curation (e.g. Svarstrom et al) and it is not clear to what extent the methods were followed from these papers.

Authors'_reply: We hereby have revised the method section by describing specific parameters for each step. For instance, we have inserted a sentence to introduce what are scaftigs and how to generate scaftigs for MAGs binning as "We first performed scaffolding of contigs using paired-end Illumina reads (SOAPdenovo v1.06) and constructed scaftigs by extracting the contiguous sequences that lack unknown bases (Ns) in each scaffold [51]." Otherwise, when referring to described methods and when no modifications were made, we prefer to direct readers to the original publication. There is no description of 'manual curation' in the revised manuscript, the reviewer might refer to CAZy (please see reply above) and for Svarstrom et al. the authors responsible for the analysis are the same on both papers and the exact identical methodology and parameters were used

There is no mention of controls, if controls were used details of these should be included.
Authors'_reply: we are not sure to understand what kind of controls the reviewer is expecting. All materials and methods used are now described in the revised manuscript.

Minor:

Supplementary figure 16 would be a lot more useful if it included the names of tools used at each stage.
Authors'_reply: Thanks for this comment. The supplementary figure 16 figure was modified as suggested.

Several figures and supplementary figures have acronyms that are not described in the figure legends or list of abbreviations.
Authors'_reply: figure legends were revised

Supplementary figure 18's legend states that combined MAGs are red, but they are green.
Authors'_reply: corrected

The abbreviations used in figures of D, FH, FL and G need to be defined in text, in abbreviations list and in the figure legends. I would also suggest that in all figures the order of groups be changed so that the dairy cow groups are next to each other and the beef cow groups are next to each other to simplify interpretation.
Authors'_reply: abbreviations used to define diets were defined at first use in the text and in each figure legend. The order of groups was modified as suggested.

Supplementary material would be easier to navigate with descriptive titles in the contents section.
Authors'_reply: Titles and subtitles are now included in the table of contents

There are several minor typos and grammatical errors throughout. The authors should review the language use in the manuscript and make corrections. Examples include but are not limited to:
Page 4: "highly nutritious protein and energy, products"
Page 11: " in accord with the normal diet of cattle normal diet" and " a hierarchical clustering analysis (Supplementary Figure 8) which that revealed"
Page 13: "25 to up 99%"
Authors'_reply: these errors were corrected in the revised version. The revised manuscript was revised for additional errors.

I believe with revisions these data will be a valuable resource.
Authors'_reply: thank you for this comment.

Reviewer #2: The study of Li and colleagues generates a novel and useful catalogue of unique rumen prokaryotc genes using deep sequencing information of 10 animals and identifying 13.8 M of non redundant genes. They also found new potential functions in rumen, particularly related to deconstruction of structural carbohydrates (CAZymes). In order to compare their data with available genomes they constructed and identified 324 MAGs (8 MAGs belonging to Prevotella genera). A large description and a useful scheme about MAGs construction is provided in methods. They made a deep and complete comparison with other available prokaryotic genes and MAGs catalogues in cattle, mouse, human and pig.
Using an independent group of 77 cows in 4 different dietary regimes they properly matched how much they improved mapped reads ratio with their new catalogue, demonstrating the advantages that this new catalogue will offer to future studies.
They also explore the effect of feed on the microbiota composition and functions in the 77 cows using ordination and procrustes rotation analysis. An interesting result found was different diets inducing differences in relative abundances rather than absence or presence of genes.
This work provides essential insights for future studies in rumen microbiome. The study is very descriptive and the main goals are well addressed. Experimental design and methods are properly chosen and described. Biological information about the new genes found is nicely discussed. Literature used is complete and adequate. While I do not find any major issue in their analysis and discussion, there are a number of errors that must be corrected. Main errors are found in Tables and Figures.

In general, numbers of Suppl. Tables not matching with their number in excel supplementary files is quite confusing. For example, Suppl. Table 13 in suppl.10, Suppl. Table 15 in suppl. 12, Suppl. Table 3 found in suppl. 2, Suppl. Table 4 found in suppl. 3, Suppl. Table 5 found in suppl. 4, Suppl. Table 7 found in suppl. 5, etc.
Authors’_reply: we are sorry for these involuntary mistakes during edition. These errors were corrected in the revised version.

Tables and Figures and the index in supplementary data file contains several errors and should be rewritten. Some titles not provided. Suppl. Table 5 and Suppl. Fig 9 are missing in the index. Besides, two last Suppl. Tables not numbered.
Authors’_reply: Titles were added to each table and figure in the table of contents. Numeration of tables and figures were corrected.

-Main Figures:
Figure 1b. Colour don't match with the description.
Authors’_reply: corrected

Figure 3. Red line for KEGG is black. MAGs instead of MGs in the legend -Suppl. Figures:
Authors’_reply: the reviewer certainly refers to Figure 4. These errors were corrected in the revised version. Thanks.

Supplementary Figure 1a, b and c. Please add a description of the diets acronyms.
Authors’_reply: done

Suppl. Figure 6: please add figures. Typing error in the title "Fonctional" instead of "Functional"
Authors’_reply: corrected

Suppl. Figure 13. Please check where complete list are available in A) B) C) and D).
Authors’_reply: all legends were modified to better indicate the diets

Suppl. Figure 18 combined is in green colour instead of red.
Authors’_reply: corrected

Suppl. Tables:
Suppl. Table 3: please, describe what does it means red cells in Sheet "913 genomes"
Authors’_reply: Red font indicates a MUMi value of > 0.54 that was used as the threshold value for species (Backhed et al. 2015; doi: 10.1016/j.chom.2015.04.004). A note was added in the excel spreadsheet.

Suppl. Table 5: please base Human abundances into 100% instead of sum 1 as you did in rumen, pig and mouse.

Authors'_reply: modified as suggested

Suppl. Table 8: Spreadsheet Holstein contains FL and FH samples instead of D and G.
Authors'_reply: thank you for pointing this out, it was a simple error in the labels that is now corrected in the revised version. This Table became Suppl. Table 11 "Suppl_Table_11_DA_KO" in the revised version.

Suppl. Table KO list in (suppl.7), CAZY in Suppl. Table 10 (suppl. 8), genera and MAGs in Suppl. Table 11 (suppl. 9) I did not found any reference in the text for Suppl. Table 14 and 16 Suppl. Table 15. Title says "317 MAGS" but might be 324. Wrongly referenced in Figures legend.
Authors'_reply: all these mismatched numbers and references were corrected in the revised version.

- Main text
Pag 4. Background. Line 9: "protein and energy products,..." instead of "protein and energy, products"
Authors'_reply: corrected

Pag 5. Data description: please, could you give some details of feed regime of the 5 Holstein and 5 Charolais animals used to create the catalogue?
Authors'_reply: this information was added in Supplementary table 14 and referred in Methods (page 18)

Pag 11. Line 1: "normal diet" written twice, "... not only in accord with the normal diet of cattle normal diet.."
Authors'_reply: corrected

Pag 24. Confusion about the total number of qualified SLGs, 575 total SLGs indicated in line 11, but two hundred and eighteen + 357 qualified summing 572 in lines 17-18.

Authors'_reply: we are not sure to understand the comment, 218 + 357= 575. Figure 16 that describes the MAGs construction process was modified and we hope the information is clearer now.

Close
