# Supplementary material for: A catalog of microbial genes from the bovine rumen unveils a specialized and diverse biomass-degrading environment
Source: Gigascience. 2020 May 30;9(6):giaa057. doi: 10.1093/gigascience/giaa057 (PMC7260996; doi:10.1093/gigascience/giaa057)
Supplement: giaa057_Response_to_Reviewer_Comments_Revision_1 [file giaa057_response_to_reviewer_comments_revision_1.pdf]

Author's Response To Reviewer Comments

Close

GIGA-D-19-00154
A catalog of microbial genes from the bovine rumen unveils a specialized and diverse biomass-degrading environment

Dear Diego,

Thank you for your email and for sharing your comparisons.

We have discussed your email and we feel a revised version may be acceptable, provided you include these basic comparisons and a description in the supplement.
The Venn diagrams are not really the extensive analysis the reviewer asked for, but may be enough to give the reader some indication regarding differences and overlap of the data.

I understand your argument regarding the other work having preprint status. You are also correct of course that the field is moving forward all the time, and comparing to other work is an ever moving target. On the other hand, the other dataset was publicly released in a repository with a stable DOI, and while the paper was a preprint at the time of submission of your work, the data itself was released in a permanent and citable form. I feel it makes sense from a reader's perspective to acknowledge this public dataset in your manuscript.

If you don't include the more detailed comparisons suggested by the reviewer, I recommend that you should be careful with your wording, for example regarding whether your work shows an expansion of available rumen genes. Such statements will need to be qualified with respect to the data that is included in the comparisons in your manuscript.

If you submit your revised manuscript, please also address the other comments of the reviewer's latest report (regarding the diverging numbers coming from SOAPdenovo and MEGAHIT, and some minor comments).

We look forward to receiving your revised submission.

Kind regards,

Hans

Dear editor,

Dear editor,

We are grateful for giving us the opportunity to revise the manuscript. All modifications made in the revised manuscript are highlighted in yellow to facilitate reading.

In order to provide a comparison with the published data set, we have now include an overall comparison between these data and ours, thereby underscoring the great diversity present in the rumen ecosystem.As requested, we modified the wording regarding the comparisons presented and statements of novelty. Also, we addressed the latest comments from the reviewer in the “Response to reviewers”

We hope that the changes made to the revised manuscript and replies to reviewers’ comments are satisfactory and the manuscript can be published in GigaScience. Do not hesitate to contact me for further clarifications.

Kind regards,

Diego Morgavi
on behalf of all authors

Reviewer report:

Reviewer #1: The authors have resubmitted their paper on a catalog of microbial genes from the bovine rumen following an initial round of review. I thank the authors for the additional work they have undertaken as requested in my previous review including assessing potential errors from the now-outdated assembly tool, carrying out additional comparisons between their MAGs and publicly available MAGs, and minor changes to methods, supplementary material and figures. While the changes to the methods, supplementary material and figures was minor I feel it has improved the readability and reproducibility.
I wish to emphasise that I do like the manuscript, the differences between groups and the overlapping functionality discussed are very interesting results. However I have some remaining concerns primarily regarding comparisons to other datasets.
The authors have added a comparison between their MAGs and the MAGs of Stewart et al., 2019, however they have not included the dataset in comparisons between CAZymes where they return to only using the smaller dataset of Stewart et al., 2018. I also can't find specific methods of this comparison included in the methods section. The smaller dataset from Stewart et al. has 69,678 CAZymes, but the larger has 442,917, which is much more comparable to the current study. The paper would benefit from consistency in the use of existing datasets.
The authors have not collapsed the protein set following UniRef guidelines at 100%, 90% and 50% similarity using their own proteins, and again using their own proteins and other known rumen proteins (e.g. from Hungate 1000, Stewart et al and others) as previously suggested. I do not believe that read mapping rates alone are sufficient to fully demonstrate the degree of novelty here. I'm sure that there *is* a huge amount of novelty here and I feel it would improve the paper to fully demonstrate this. The abstract states "The catalog expands by several folds the dataset of carbohydrate-degrading enzymes described in the rumen." which cannot be supported with the current analysis as direct comparisons with all available rumen CAZymes have not been done. Similarly the paragraph discussing the results from figure 1 on page 6 talks about expanding the rumen catalog without considering other available datasets- this study has improved on the Hess et al dataset several fold, and certainly will have expanded the available rumen genes, but compared to all available rumen genes how much novelty is there?

AUTHORS REPLY:
We agree that for statements like “expands by several folds … the rumen dataset”, we should compare this work with all available information. This comparison (and showing or not the exact amount of new genes) is not the main message of the work and we feel frustrated that it has taken this much questioning from the reviewer. We think that a complete comparison brings little, if any, originality to the manuscript. In addition to the considerable amount of time and resources needed, making more complex comparisons would be like chasing a moving target.
In the revised-II manuscript, we compared our dataset with the latest Steward et al. dataset using Diamond blastp (at 100, 90 and 50%) with a display of overlapping and unique proteins in both datasets. This type of comparison gives a glimpse of the novelty but particularly of the yet uncharted diversity present in the rumen ecosystem.
The methodology used to compare CAZymes was added in the Methods section, ‘Gene catalog annotation’ sub-heading
To avoid confusion, we modified the sentence in the abstract and all similar statements of comparative sizing.

Given the circumstances of the project beginning before SOAPdenovo's authors recommended against using it on microbiomes and given that the authors demonstrate on a subset of the data that most of these are assembled by newer tools I accept that the majority of the genes they have assembled are likely to be accurate and the main limitation of the tool is that fewer genes have assembled. It is a shame that these data could potentially produce an even larger set of genes with a more modern assembler, but redoing the analysis would be extreme given the amount of downstream analyses in the manuscript. That being said, I don't fully understand part of the response:

"...853,085 genes (50.13%) assembled by SOAPdenovo v1.06 were non-redundant genes and 737,617

of these genes were also assembled by MEGAHIT v2.19. Most importantly, 842,886 genes assembled by SOAPdenovo v1.06 were also present in the MEGAHIT v2.19 dataset (Rebuttal Table 2). Thus, 92.88% of genes [(737,617 + 842,886) / 1,701,641] assembled by our original SOAPdenovo pipeline were identified by the MEGAHIT pipeline (showing ≥95% identity and ≥90% coverage)."

Here the authors have presented two different numbers for genes assembled by both SOAPdenovo and MEGAHIT2, a non-redundant set (737,617) and another number (842,886), and then use the sum of these to determine the percentage of SOAPdenovo assembled genes that were assembled by both tools. It is not clear to me that these two sets do not overlap. I apologise if I am misunderstanding the results, I'm afraid the table isn't making it much clearer. Can you please clarify the difference between these two so I can better understand where the 92.88% figure came from.

AUTHORS REPLY:
We are sorry if this was unclear. The table shows that:

1) The total number of assembled genes for sample Ann 552 by MEGAHIT v2.19 was 2,429,342; and those assembled by SOAPdenovo v1.06 was 1,701,641.

2) By using CD-HIT, we generated a non-redundant gene set, which was composed of 1,599,864 genes assembled by MEGAHIT v2.19 and 853,085 genes assembled by SOAPdenovo v1.06.

3) From the non-redundant sub-set of 853,085 genes originating from SOAPdenovo, 737,617 showed high identity (≥95%) with the total number of assembled MEGAHIT genes and were thus present in this latter dataset.

4) From the non-redundant sub-set of 1,599,864 genes originating from MEGAHIT, 842,886 showed high identity (≥95%) with the total number of assembled SOAPdenovo genes and represented those genes.

5) Thus, 92.88% of genes [(737,617 + 842,886) / 1,701,641] assembled by our original SOAPdenovo pipeline were also identified by the MEGAHIT pipeline (showing ≥95% identity and ≥90% coverage).

I feel figures 1a+b need updating as they show the overlap between the genes in this study and the genes from one other dataset, the smallest dataset discussed in the rest of the paper, I would prefer this to show this study's genes vs all rumen datasets discussed either all pooled together or individually.

AUTHORS REPLY:
This is related to the first comment. Please refer to our reply above. In accord with the remark, the comparison with the dataset of Hess et al. (Fig 1 a, b) was moved in the supplementary material. Figure 1c from the previous version is now the only graph shown in the revised-II manuscript.

Minor:

On page 6 I believe there is something missing from the brackets here, a citation?: "We identified 324 MAGs with an average size of 1.8 Mbp (minimum threshold of 1 Mbp; see for more information on these MAGs)."

AUTHORS REPLY: thanks, a reference to a supplementary table was missing (supplementary Table 15 in the previous version of the manuscript). This was corrected in the revised-II manuscript and subsequent table numbers were modified.

Page 12: "25% to up to 99%" should change to "25% to 99%"

AUTHORS REPLY: change made

On page 6 please define medium-quality as you have defined high-quality I noticed that the assembly statistics for the full dataset from SOAPdenovo are not included, could these be added to the supplement?

AUTHORS REPLY: the definition for medium quality MAGs was added to the manuscript. As requested, the assembly statistics for the 10 samples submitted to deep sequencing was added to Supplementary Table 4.

Close
