# Supplementary material for: A catalog of microbial genes from the bovine rumen unveils a specialized and diverse biomass-degrading environment
Source: Gigascience. 2020 May 30;9(6):giaa057. doi: 10.1093/gigascience/giaa057 (PMC7260996; doi:10.1093/gigascience/giaa057)
Supplement: giaa057_Response_to_Reviewer_Comments_Revision_2 [file giaa057_response_to_reviewer_comments_revision_2.pdf]

Author's Response To Reviewer Comments

Close

Dear Editor,

We are really pleased that the manuscript is accepted for publication. Thank you for handling this submission in such a good manner.
Below are the reply to comments and other changes made to the manuscript. All changes are highlighted in yellow in the revised manuscript.

1) Our data curator will contact you to prepare the supporting dataset for our repository GigaDB. To aid reproducibility, we will need some more information on the data, and maybe different formats, such as tabular data in csv format. Our data curator will contact you soon with details.

REPLY: we provided Chris Hunter with tables in machine-readable format and other information that he required.

2) Please revise the new supplemental Figure 1e. The percentage overlap in this venn-style diagram is mis-represented, visually, as the 15.5% overlap looks exactly the same as the 91.5% overlap. Please either change the diagram or replace it with a simple table.

REPLY: the diagram was changed using proportions as requested.

3) p20: is there a mistake in the text? "The *four* public rumen microbial datasets used in this study include..." Five datasets are described here, it seems?

REPLY: the mistake was corrected. Thanks

4) Once you get the citation details for the supporting GigaDB dataset, please include a citation to this dataset (including the DOI link) to your reference list, and cite this reference in the data availability section and elsewhere in the manuscript, where appropriate.

REPLY: The dataset is now cited in the manuscript and included in the reference list.

OTHER CHANGES

- Supplementary Table 14 in the previous version had redundant information with Supplementary Table 4. All information was merged in Supplementary Table 4 of the revised-3 manuscript. Former Supplementary Tables 15, 16 and 17 were renumbered to Suppl. Tables 14, 15 and 16 in the revised-3 manuscript.
-
- GigaScience DB curator, Chris Hunter asked for some methodological clarifications for Supplementary Table 13. These changes are highlighted in yellow on pages 16 and 28.
-
- In Supplementary Figures and Tables document, minor modifications related to changes mentioned above, e.g. Table numbering, are highlighted.

Close
