# Supplementary material for: A catalog of microbial genes from the bovine rumen unveils a specialized and diverse biomass-degrading environment
Source: Gigascience. 2020 May 30;9(6):giaa057. doi: 10.1093/gigascience/giaa057 (PMC7260996; doi:10.1093/gigascience/giaa057)
Supplement: giaa057_Reviewer_1_Report_Original_Submission — Amanda Warr -- 6/19/2019 Reviewed [file giaa057_reviewer_1_report_original_submission.pdf]

Reviewer Report

Title: A catalog of microbial genes from the bovine rumen unveils a specialized and diverse biomass-degrading environment

Version: Original Submission **Date:** 6/19/2019

Reviewer name: Amanda Warr

Reviewer Comments to Author:

The authors present a collection of microbial genes from the bovine rumen. Accurate gene catalogs are an important resource for an environment that is largely underrepresented in databases and the rumen is a particularly interesting environment. They also present data on differences in microbiome composition, abundance of different species and microbial gene functions between cows from different genetic backgrounds fed on different diets. While the paper is interesting and the resources produced are likely important I have some concerns.

The authors use SOAPdenovo v1.06 for their assemblies. I question the appropriateness of this choice given SOAPdenovo's documentation recommends MEGAHIT, a tool designed to handle metagenomic data which SOAPdenovo is not designed for. The paper for MEGAHIT, by the same authors as SOAPdenovo, also states "Note that SOAPdenovo2 and Minia are designed to assemble a single genome. For metagenomic data, which involve numerous different genomes with uneven depth coverage and cross-genome repeats, specifically designed algorithms are required to achieve good assembly quality." There has also been a more recent version of SOAPdenovo than the one used, SOAPdenovo2. Additionally, other tools specifically designed for metagenomic assembly have been available for a number of years, e.g. IDBA-UD. It would have been more appropriate to use a tool designed for metagenomic assembly. My concern is that older tools don't tend to perform as well and a tool designed to work on a single genome may have produced false joins in the contigs which in turn could lead to some false gene predictions and truncations. What is the justification for using this tool?

The authors compare their results to 2 other publicly available datasets, Stewart et al., 2018 and Hess et al., 2011 and demonstrate novelty in their data compared to the other two, however beyond mapping rates the method of comparison (e.g. for %IDY) is not clear, nor is it clear what tool was used for mapping. Please add a section to the methods specifically describing methods for comparisons with other datasets and specify in results sections which analysis lead to the result. Stewart et al. have recently produced a much more extensive dataset (https://www.biorxiv.org/content/10.1101/489443v1, data available: https://datashare.is.ed.ac.uk/handle/10283/3224) producing almost 5000 MAGs. Additionally there are other rumen datasets available which should be included to give a complete picture of the novelty in the authors' dataset and give some idea of the extent of novelty still unexplored in the rumen, e.g. Parks et al., Solden et al. and Svarstrom et al. The comparisons carried out by the authors focus on similarities between MAGs and similarities between CAZymes, however the comparison between the full set of proteins the authors identified and previously identified proteins is minimal. The authors have used CD-HIT once on their own data to remove redundant genes and once with a set of Hess et al.'s genes, but I

suggest the authors collapse the protein set following UniRef guidelines at 100%, 90% and 50% similarity using their own proteins, and again using their own proteins and all other known rumen proteins to fully demonstrate novelty and reduce redundancy. This should be done at least for Hungate 1000 and the largest dataset of Stuart et al., but would preferably include other datasets. There is mention that this was partly done for the Hess et al. dataset, however the methods described are not detailed or reproducible"13.83 M genes from current study and 2.46 M genes from JGI were pooled together to identify shared genes using CD-HIT".
Recommended CD-HIT parameters:
ID=100%: -c 1.0
ID=90%: -c 0.90 -n 5 -s 0.80
ID=50%: -c 0.50 -n 3 -s 0.80
(using output of previous level as input of lower level)
Following this the authors can report the number of clusters in their dataset that are novel relative to the other datasets
The authors identify CAZymes by comparing predicted proteins to the CAZy database and to Hidden Markov models built from each CAZy family. Ideally, a tool such as dbCAN2 should be used to annotate CAZymes. This tool is automated and uses multiple methods to annotate CAZymes and is generally more accurate than using one method. Ideally all genes should be annotated with KEGG (which was used) and Uniref (which was not).
Parts of the methods section are a little difficult to follow, for example the first mention of scaftigs is on page 23 and it is not immediately clear these have come from the assembly on page 21. Suggest the title of section on page 21 include mention of scaftigs (i.e. Construction of the rumen microbial scaftigs and gene catalog) and/or including the details of the assembly tool again in the section on page 23. The authors should review the methods and ensure that it is clear where the data used for each section originated. Additionally the authors stated multiple times that methods were "as described previously" or similar, it would be helpful to have more of a description so there is less need to go searching for the methods and more ease of reproducibility, even if this was just added to the supplement. Additionally, in some cases the papers used describe specific parameters or describe manual curation (e.g. Svarstrom et al) and it is not clear to what extent the methods were followed from these papers.
There is no mention of controls, if controls were used details of these should be included.
Minor:
Supplementary figure 16 would be a lot more useful if it included the names of tools used at each stage.
Several figures and supplementary figures have acronyms that are not described in the figure legends or list of abbreviations.
Supplementary figure 18's legend states that combined MAGs are red, but they are green.
The abbreviations used in figures of D, FH, FL and G need to be defined in text, in abbreviations list and in the figure legends. I would also suggest that in all figures the order of groups be changed so that the dairy cow groups are next to each other and the beef cow groups are next to each other to simplify interpretation.
Supplementary material would be easier to navigate with descriptive titles in the contents section.
There are several minor typos and grammatical errors throughout. The authors should review the language use in the manuscript and make corrections. Examples include but are not limited to:

Page 4: "highly nutritious protein and energy, products"
Page 11: " in accord with the normal diet of cattle normal diet" and " a hierarchical clustering analysis (Supplementary Figure 8) which that revealed"
Page 13: "25 to up 99%"
I believe with revisions these data will be a valuable resource.

Methods

Are the methods appropriate to the aims of the study, are they well described, and are necessary controls included? Choose an item.

Conclusions

Are the conclusions adequately supported by the data shown? Choose an item.

Reporting Standards

Does the manuscript adhere to the journal's guidelines on minimum standards of reporting? Choose an item.

Choose an item.

Statistics

Are you able to assess all statistics in the manuscript, including the appropriateness of statistical tests used? Choose an item.

Quality of Written English

Please indicate the quality of language in the manuscript: Choose an item.

Declaration of Competing Interests

Please complete a declaration of competing interests, considering the following questions:

- Have you in the past five years received reimbursements, fees, funding, or salary from an organisation that may in any way gain or lose financially from the publication of this manuscript, either now or in the future?
- Do you hold any stocks or shares in an organisation that may in any way gain or lose financially from the publication of this manuscript, either now or in the future?
- Do you hold or are you currently applying for any patents relating to the content of the manuscript?
- Have you received reimbursements, fees, funding, or salary from an organization that holds or has applied for patents relating to the content of the manuscript?
- Do you have any other financial competing interests?

- Do you have any non-financial competing interests in relation to this paper?

If you can answer no to all of the above, write 'I declare that I have no competing interests' below. If your reply is yes to any, please give details below.

I declare that I have no competing interests

I agree to the open peer review policy of the journal. I understand that my name will be included on my report to the authors and, if the manuscript is accepted for publication, my named report including any attachments I upload will be posted on the website along with the authors' responses. I agree for my report to be made available under an Open Access Creative Commons CC-BY license (http://creativecommons.org/licenses/by/4.0/). I understand that any comments which I do not wish to be included in my named report can be included as confidential comments to the editors, which will not be published.

Choose an item.

To further support our reviewers, we have joined with Publons, where you can gain additional credit to further highlight your hard work (see: https://publons.com/journal/530/gigascience). On publication of this paper, your review will be automatically added to Publons, you can then choose whether or not to claim your Publons credit. I understand this statement.

Yes Choose an item.
