# Supplementary material for: A catalog of microbial genes from the bovine rumen unveils a specialized and diverse biomass-degrading environment
Source: Gigascience. 2020 May 30;9(6):giaa057. doi: 10.1093/gigascience/giaa057 (PMC7260996; doi:10.1093/gigascience/giaa057)
Supplement: giaa057_Reviewer_1_Report_Revision_1 — Amanda Warr -- 12/10/2019 Reviewed [file giaa057_reviewer_1_report_revision_1.pdf]

Reviewer Report

Title: A catalog of microbial genes from the bovine rumen unveils a specialized and diverse biomass-degrading environment

Version: Revision 1 **Date:** 12/10/2019

Reviewer name: Amanda Warr

Reviewer Comments to Author:

The authors have resubmitted their paper on a catalog of microbial genes from the bovine rumen following an initial round of review. I thank the authors for the additional work they have undertaken as requested in my previous review including assessing potential errors from the now-outdated assembly tool, carrying out additional comparisons between their MAGs and publicly available MAGs, and minor changes to methods, supplementary material and figures. While the changes to the methods, supplementary material and figures was minor I feel it has improved the readability and reproducibility. I wish to emphasise that I do like the manuscript, the differences between groups and the overlapping functionality discussed are very interesting results. However I have some remaining concerns primarily regarding comparisons to other datasets.

The authors have added a comparison between their MAGs and the MAGs of Stewart et al., 2019, however they have not included the dataset in comparisons between CAZymes where they return to only using the smaller dataset of Stewart et al., 2018. I also can't find specific methods of this comparison included in the methods section. The smaller dataset from Stewart et al. has 69,678 CAZymes, but the larger has 442,917, which is much more comparable to the current study. The paper would benefit from consistency in the use of existing datasets.

The authors have not collapsed the protein set following UniRef guidelines at 100%, 90% and 50% similarity using their own proteins, and again using their own proteins and other known rumen proteins (e.g. from Hungate 1000, Stewart et al and others) as previously suggested. I do not believe that read mapping rates alone are sufficient to fully demonstrate the degree of novelty here. I'm sure that there *is* a huge amount of novelty here and I feel it would improve the paper to fully demonstrate this. The abstract states "The catalog expands by several folds the dataset of carbohydrate-degrading enzymes described in the rumen." which cannot be supported with the current analysis as direct comparisons with all available rumen CAZymes have not been done. Similarly the paragraph discussing the results from figure 1 on page 6 talks about expanding the rumen catalog without considering other available datasets- this study has improved on the Hess et al dataset several fold, and certainly will have expanded the available rumen genes, but compared to all available rumen genes how much novelty is there?

Given the circumstances of the project beginning before SOAPdenovo's authors recommended against using it on microbiomes and given that the authors demonstrate on a subset of the data that most of these are assembled by newer tools I accept that the majority of the genes they have assembled are likely to be accurate and the main limitation of the tool is that fewer genes have assembled. It is a shame that these data could potentially produce an even larger set of genes with a more modern

assembler, but redoing the analysis would be extreme given the amount of downstream analyses in the manuscript. That being said, I don't fully understand part of the response:
"...853,085 genes (50.13%) assembled by SOAPdenovo v1.06 were non-redundant genes and 737,617 of these genes were also assembled by MEGAHIT v2.19. Most importantly, 842,886 genes assembled by SOAPdenovo v1.06 were also present in the MEGAHIT v2.19 dataset (Rebuttal Table 2). Thus, 92.88% of genes [(737,617 + 842,886) / 1,701,641] assembled by our original SOAPdenovo pipeline were identified by the MEGAHIT pipeline (showing â‰¥95% identity and â‰¥90% coverage)."
Here the authors have presented two different numbers for genes assembled by both SOAPdenovo and MEGAHIT2, a non-redundant set (737,617) and another number (842,886), and then use the sum of these to determine the percentage of SOAPdenovo assembled genes that were assembled by both tools. It is not clear to me that these two sets do not overlap. I apologise if I am misunderstanding the results, I'm afraid the table isn't making it much clearer. Can you please clarify the difference between these two so I can better understand where the 92.88% figure came from.
I feel figures 1a+b need updating as they show the overlap between the genes in this study and the genes from one other dataset, the smallest dataset discussed in the rest of the paper, I would prefer this to show this study's genes vs all rumen datasets discussed either all pooled together or individually.
Minor:
On page 6 I believe there is something missing from the brackets here, a citation?: "We identified 324 MAGs with an average size of 1.8 Mbp (minimum threshold of 1 Mbp; see for more information on these MAGs)."
Page 12: "25% to up to 99%" should change to "25% to 99%"
On page 6 please define medium-quality as you have defined high-quality
I noticed that the assembly statistics for the full dataset from SOAPdenovo are not included, could these be added to the supplement?

Methods

Are the methods appropriate to the aims of the study, are they well described, and are necessary controls included? Choose an item.

Conclusions

Are the conclusions adequately supported by the data shown? Choose an item.

Reporting Standards

Does the manuscript adhere to the journal’s guidelines on minimum standards of reporting? Choose an item.

Choose an item.

Statistics

Are you able to assess all statistics in the manuscript, including the appropriateness of statistical tests used? Choose an item.

Quality of Written English

Please indicate the quality of language in the manuscript: Choose an item.

Declaration of Competing Interests

Please complete a declaration of competing interests, considering the following questions:

- Have you in the past five years received reimbursements, fees, funding, or salary from an organisation that may in any way gain or lose financially from the publication of this manuscript, either now or in the future?
- Do you hold any stocks or shares in an organisation that may in any way gain or lose financially from the publication of this manuscript, either now or in the future?
- Do you hold or are you currently applying for any patents relating to the content of the manuscript?
- Have you received reimbursements, fees, funding, or salary from an organization that holds or has applied for patents relating to the content of the manuscript?
- Do you have any other financial competing interests?
- Do you have any non-financial competing interests in relation to this paper?

If you can answer no to all of the above, write 'I declare that I have no competing interests' below. If your reply is yes to any, please give details below.

I declare that I have no competing interests

I agree to the open peer review policy of the journal. I understand that my name will be included on my report to the authors and, if the manuscript is accepted for publication, my named report including any attachments I upload will be posted on the website along with the authors' responses. I agree for my report to be made available under an Open Access Creative Commons CC-BY license (http://creativecommons.org/licenses/by/4.0/). I understand that any comments which I do not wish to be included in my named report can be included as confidential comments to the editors, which will not be published.

Choose an item.

To further support our reviewers, we have joined with Publons, where you can gain additional credit to further highlight your hard work (see: https://publons.com/journal/530/gigascience). On publication of this paper, your review will be automatically added to Publons, you can then choose whether or not to claim your Publons credit. I understand this statement.

Yes Choose an item.
