# Supplementary material for: A catalog of microbial genes from the bovine rumen unveils a specialized and diverse biomass-degrading environment
Source: Gigascience. 2020 May 30;9(6):giaa057. doi: 10.1093/gigascience/giaa057 (PMC7260996; doi:10.1093/gigascience/giaa057)
Supplement: giaa057_Reviewer_2_Report_Original_Submission — Marina Martinez Alvaro, PhD -- 12/10/2019 Reviewed [file giaa057_reviewer_2_report_original_submission.pdf]

Reviewer Report

Title: A catalog of microbial genes from the bovine rumen unveils a specialized and diverse biomass-degrading environment

Version: Original Submission **Date:** 12/10/2019

Reviewer name: Marina Martinez Alvaro, PhD

Reviewer Comments to Author:

The study of Li and colleagues generates a novel and useful catalogue of unique rumen prokaryotc genes using deep sequencing information of 10 animals and identifying 13.8 M of non redundant genes. They also found new potential functions in rumen, particularly related to deconstruction of structural carbohydrates (CAZymes). In order to compare their data with available genomes they constructed and identified 324 MAGs (8 MAGs belonging to Prevotella genera). A large description and a useful scheme about MAGs construction is provided in methods. They made a deep and complete comparison with other available prokaryotic genes and MAGs catalogues in cattle, mouse, human and pig.
Using an independent group of 77 cows in 4 different dietary regimes they properly matched how much they improved mapped reads ratio with their new catalogue, demonstrating the advantages that this new catalogue will offer to future studies.
They also explore the effect of feed on the microbiota composition and functions in the 77 cows using ordination and procrustes rotation analysis. An interesting result found was different diets inducing differences in relative abundances rather than absence or presence of genes.
This work provides essential insights for future studies in rumen microbiome. The study is very descriptive and the main goals are well addressed. Experimental design and methods are properly chosen and described. Biological information about the new genes found is nicely discussed. Literature used is complete and adequate. While I do not find any major issue in their analysis and discussion, there are a number of errors that must be corrected. Main errors are found in Tables and Figures.
In general, numbers of Suppl. Tables not matching with their number in excel supplementary files is quite confusing. For example, Suppl. Table 13 in suppl.10, Suppl. Table 15 in suppl. 12, Suppl. Table 3 found in suppl. 2, Suppl. Table 4 found in suppl. 3, Suppl. Table 5 found in suppl. 4, Suppl. Table 7 found in suppl. 5, etc.
Tables and Figures and the index in supplementary data file contains several errors and should be rewritten. Some titles not provided. Suppl. Table 5 and Suppl. Fig 9 are missing in the index. Besides, two last Suppl. Tables not numbered.
-Main Figures:
Figure 1b. Colour don't match with the description.
Figure 3. Red line for KEGG is black. MAGs instead of MGs in the legend
-Suppl. Figures:
Supplementary Figure 1a, b and c. Please add a description of the diets acronyms.
Suppl. Figure 6: please add figures. Typing error in the title "Fonctional" instead of "Functional"
Suppl. Figure 13. Please check where complete list are available in A) B) C) and D).

Suppl. Figure 18 combined is in green colour instead of red.
Suppl. Tables:
Suppl. Table 3: please, describe what does it means red cells in Sheet "913 genomes"
Suppl. Table 5: please base Human abundances into 100% instead of sum 1 as you did in rumen, pig and mouse.
Suppl. Table 8: Spreadsheet Holstein contains FL and FH samples instead of D and G.
Suppl. Table KO list in (suppl.7), CAZY in Suppl. Table 10 (suppl. 8), genera and MAGs in Suppl. Table 11 (suppl. 9)
I did not found any reference in the text for Suppl. Table 14 and 16
Suppl. Table 15. Title says "317 MAGS" but might be 324. Wrongly referenced in Figures legend.

- Main text

Pag 4. Background. Line 9: "protein and energy products,…" instead of "protein and energy, products"
Pag 5. Data description: please, could you give some details of feed regime of the 5 Holstein and 5 Charolais animals used to create the catalogue?
Pag 11. Line 1: "normal diet" written twice, "… not only in accord with the normal diet of cattle normal diet.."
Pag 24. Confusion about the total number of qualified SLGs, 575 total SLGs indicated in line 11, but two hundred and eighteen + 357 qualified summing 572 in lines 17-18.

Methods

Are the methods appropriate to the aims of the study, are they well described, and are necessary controls included? Choose an item.

Conclusions

Are the conclusions adequately supported by the data shown? Choose an item.

Reporting Standards

Does the manuscript adhere to the journal's guidelines on minimum standards of reporting? Choose an item.

Choose an item.

Statistics

Are you able to assess all statistics in the manuscript, including the appropriateness of statistical tests used? Choose an item.

Quality of Written English

Please indicate the quality of language in the manuscript: Choose an item.

Declaration of Competing Interests

Please complete a declaration of competing interests, considering the following questions:

- Have you in the past five years received reimbursements, fees, funding, or salary from an organisation that may in any way gain or lose financially from the publication of this manuscript, either now or in the future?
- Do you hold any stocks or shares in an organisation that may in any way gain or lose financially from the publication of this manuscript, either now or in the future?
- Do you hold or are you currently applying for any patents relating to the content of the manuscript?
- Have you received reimbursements, fees, funding, or salary from an organization that holds or has applied for patents relating to the content of the manuscript?
- Do you have any other financial competing interests?
- Do you have any non-financial competing interests in relation to this paper?

If you can answer no to all of the above, write 'I declare that I have no competing interests' below. If your reply is yes to any, please give details below.

I declare that I have no competing interests

I agree to the open peer review policy of the journal. I understand that my name will be included on my report to the authors and, if the manuscript is accepted for publication, my named report including any attachments I upload will be posted on the website along with the authors' responses. I agree for my report to be made available under an Open Access Creative Commons CC-BY license (http://creativecommons.org/licenses/by/4.0/). I understand that any comments which I do not wish to be included in my named report can be included as confidential comments to the editors, which will not be published.

Choose an item.

To further support our reviewers, we have joined with Publons, where you can gain additional credit to further highlight your hard work (see: https://publons.com/journal/530/gigascience). On publication of this paper, your review will be automatically added to Publons, you can then choose whether or not to claim your Publons credit. I understand this statement.

Yes Choose an item.
